# Interplay between WNT/PI3K-mTOR axis and the microbiota in APC-driven colorectal carcinogenesis: data from a pilot study and possible implications for CRC prevention

**DOI:** 10.1186/s12967-024-05305-5

**Published:** 2024-07-05

**Authors:** Floriana Jessica Di Paola, Chiara Alquati, Gabriele Conti, Giulia Calafato, Silvia Turroni, Federica D’Amico, Claudio Ceccarelli, Francesco Buttitta, Alice Bernardi, Dajana Cuicchi, Gilberto Poggioli, Daniela Turchetti, Simona Ferrari, Renato Cannizzaro, Stefano Realdon, Patrizia Brigidi, Luigi Ricciardiello

**Affiliations:** 1 IRCCS Azienda Ospedaliero-Universitaria di Bologna, Bologna, Italy; 2 Department of Medical and Surgical Sciences, University of Bologna, Bologna, Italy; 3 Department of Pharmacy and Biotechnology, University of Bologna, Bologna, Italy; 4 Oncological Gastroenterology, Centro di Riferimento Oncologico di Aviano (CRO) IRCCS, Aviano, Italy; 5 Department of Medical, Surgical and Health Sciences, University of Trieste, Trieste, Italy; 6 Centre for Applied Biomedical Research (CRBA), University of Bologna, Bologna, Italy

**Keywords:** Colorectal cancer, Familial adenomatous polyposis, Wnt/β-catenin, PI3K/mTOR, Microbiota

## Abstract

**Background:**

Wnt/β-catenin signalling impairment accounts for 85% of colorectal cancers (CRCs), including sporadic and familial adenomatous polyposis (FAP) settings. An altered PI3K/mTOR pathway and gut microbiota also contribute to CRC carcinogenesis. We studied the interplay between the two pathways and the microbiota composition within each step of CRC carcinogenesis.

**Methods:**

Proteins and target genes of both pathways were analysed by RT-qPCR and IHC in tissues from healthy faecal immunochemical test positive (FIT+, *n* = 17), FAP (*n* = 17) and CRC (*n* = 15) subjects. CRC-related mutations were analysed through NGS and Sanger. Oral, faecal and mucosal microbiota was profiled by 16 S rRNA-sequencing.

**Results:**

We found simultaneous hyperactivation of Wnt/β-catenin and PI3K/mTOR pathways in FAP-lesions compared to CRCs. Wnt/β-catenin molecular markers positively correlated with *Clostridium_sensu_stricto_1* and negatively with *Bacteroides* in FAP faecal microbiota. *Alistipes*, *Lachnospiraceae*, and *Ruminococcaceae* were enriched in FAP stools and adenomas, the latter also showing an overabundance of *Lachnoclostridium*, which positively correlated with *cMYC*. In impaired-mTOR-mutated CRC tissues, p-S6R correlated with *Fusobacterium* and *Dialister*, the latter also confirmed in the faecal-ecosystem.

**Conclusions:**

Our study reveals an interplay between Wnt/β-catenin and PI3K/mTOR, whose derangement correlates with specific microbiota signatures in FAP and CRC patients, and identifies new potential biomarkers and targets to improve CRC prevention, early adenoma detection and treatment.

**Supplementary Information:**

The online version contains supplementary material available at 10.1186/s12967-024-05305-5.

## Introduction

Approximately 85% of colorectal cancers (CRCs) develop on a background of Wnt/β-catenin signalling impairment [1]. Levels of β-catenin, a downstream effector of the canonical Wnt signalling, are negatively regulated by the tumor suppressor protein adenomatous polyposis coli (APC). Genetic alterations in the Wnt/β-catenin pathway components, such as *APC* gene mutations, result in the intrinsic aberrant canonical activation of the pathway due to β-catenin cytosolic accumulation and nuclear translocation with consequent upregulation of downstream targets (i.e. *AXIN2*, *cMYC*, *CCND1*) [2–5].

Germline or somatic loss-of-function variants in the *APC* gene are critical for CRC onset. Inherited *APC* gene pathogenic variants are responsible for familial adenomatous polyposis (FAP) with up to a 100% risk of developing CRC during a person’s lifetime [6].

Several studies have described PI3K/AKT/mTOR (also referred to as PI3K/mTOR) derangement, frequently caused by genetic alterations of key genes such as *PIK3CA*, *PTEN* and *AKT*, in *APC*-driven intestinal tumorigenesis, highlighting the relevance of the WNT-mTOR interplay [7, 8]. Once activated, mTOR mediates the phosphorylation of its two downstream targets: the eukaryotic translation initiation factor 4E binding protein 1 (4E-BP1) and p70S6 ribosomal kinase 1 (p70S6K), which lead to ribosomal protein S6 phosphorylation (p-S6R) and consequent RNA translation induction [9, 10].

Because of their involvement in CRC carcinogenesis, the Wnt/β-catenin and PI3K/mTOR signalling represent attractive targets for chemoprevention and treatment [11, 12]. Importantly, each pathway is deregulated by altered gut microbiota composition, above all by virulence factors of pathogens or pathobionts [13–16], with an impact on cancer development, progression and treatment response [17, 18].

This study aims to identify and compare the specific signatures of healthy faecal immunochemical test-positive subjects (FIT+), FAP and CRC patients, correlating Wnt/β-catenin and PI3K/mTOR derangement with oral, faecal and mucosa-associated microbial dysbiosis. The aim is to identify potential molecular biomarkers and/or targets for the prevention, early detection, and treatment of premalignant lesions.

To the best of our knowledge, no previous study has performed such a comprehensive and comparative profiling.

## Materials and methods

### Patient samples

Seventeen unoperated FAP (aged 19–57 years) and 15 CRC patients (aged 51–80 years) were enrolled together with 17 FIT + subjects (aged 50–70 years) who tested negative during screening colonoscopy. Oral swabs, mucosal biopsies and stools were collected from the enrolled patients. Fresh biopsies were collected from FIT+, FAP normal appearing colonic mucosa (NM) and FAP adenomas (P; size < 5 mm) during colonoscopy of the sigmoid colon. Surgical resected colonic tissues (NM and CRC) were collected from patients with sporadic CRC. Non-cancerous mucosa (NM) was resected at a minimum distance of 5 cm from the tumour. More details are described in the Supplementary Methods.

### Histology and immunohistochemical analysis

Tissue samples were formalin-fixed and paraffin-embedded. For β-catenin, phospho-p70 S6 Kinase (p-p70S6K) and phospho-S6 ribosomal protein (p-S6R), slides were stained following the DAB15’ protocol using a Leica BOND RX automated immunostainer (Leica Biosystems). For Ki67 antigen retrieval was performed with a citrate buffer (pH 6.0) at 120 ºC for 20 min and the slides were incubated with the antibody overnight at 4 ºC. Slides were incubated with the following antibodies: total β-catenin (clone 14, BD Biosciences; dilution 1:2,500), p-p70S6K (Thr389, Thr412) (PA5-104842, Invitrogen; dilution 1:100), p-S6R (Ser235/236) (#2211 Cell Signaling Technology; dilution 1:600), Ki67 (clone 8D5, #9449 Cell Signaling Technology; dilution 1:700). Slides were scanned using a Leica ICC50 W microscope (Leica Biosystems) for quantification. The Ki67 proliferation index was expressed as the ratio between positive nuclei and total number of nuclei per crypt analysed on 6 to 10 full-length, well-orientated, longitudinal crypts. β-catenin (nuclear and cytoplasmatic), p-p70S6K and p-S6R extent of staining was graded according to the following scales: (0 = none, 1 = < 25%, 2 = 25–50%, 3 = 50–75%, 4 = 75–100%), while staining intensity was graded using a scale 0–3 as follows: (0 = negative, 1 = weak, 2 = intermediate and 3 = strong). The intensity and extent scores were multiplied to generate the immunoreactivity score (IS; range, 0–12) for each case. Data were normalized on FIT + median except for nuclear β-catenin and p-p70S6K because the median value of FIT + was zero. Frequency classes (negative – low – medium – high) were created for the extent of the IS. Details of the relative frequency distribution calculation are described in the Supplementary Methods.

### RNA extraction and qRT-PCR

Total RNA was extracted with ice-cold TRIzol® reagent (Invitrogen™; Thermo Fisher Scientific) according to the manufacturer’s instructions. RNA quantification and purity were evaluated with the NanoDrop 1000 Spectrophotometer. Two µg of total RNA was reverse transcribed using the High-capacity cDNA Reverse Transcription Kit with RNase Inhibitor according to the manufacturer’s protocol (#4,374,966, Applied Biosystems™; Thermo Fisher Scientific). qPCR was performed on a QuantStudio5 thermal cycler (Applied Biosystems™; Thermo Fisher Scientific) using the TaqMan Fast Advanced Master Mix (Applied Biosystems™; Thermo Fisher Scientific) and the Taqman gene expression assays (Thermo Fisher Scientific) for *AXIN2* (Assay ID: Hs00610344_m1), *cMYC* (Assay ID: Hs00153408_m1), *CCND1* (Assay ID: Hs00765553_m1), *LGR5* (Assay ID: Hs00969422_m1), *RPS6* (Assay ID: Hs03044100_g1), *VEGFA* (Assay ID: Hs00900055_m1), *TP53* (Assay ID: Hs01034249_m1), *ACTB* (Assay ID: Hs99999903_m1), *GAPDH* (Assay ID: Hs03929097_g1). *GAPDH* and *ACTB* were used as reference genes for the normalization of qPCR data. Fold change values were calculated using the 2^−ΔΔCt^ method. All FAP and CRC relative fold changes were normalized to FIT + controls; FAP adenomas and CRC relative fold changes were normalized to the respective normal mucosal tissues.

### DNA extraction and next-generation sequencing

Genomic DNA was extracted from snap frozen tissues using the Maxwell 16 instrument (Promega Corporation). Next-generation sequencing (NGS) was performed on an Ion Chef and Ion Gene Studio S5 System (Ion Torrent, Thermo Fisher Scientific) using the Ion Ampliseq Cancer HotSpot Panel v2 and a custom Ion AmpliSeq On-Demand panel (Thermo Fisher Scientific), designed to detect SNV and small indel variants in 21 genes associated with cancer including the *APC* gene. Briefly, genomic DNA was quantified using a NanoDrop 1000 Spectrophotometer and 10–20 ng of DNA was used to manually prepare libraries with Ion AmpliSeq Library Kit Plus and IonXpress Barcode Adapter Kit. The barcoded and purified libraries were quantified using the Ion Library TaqMan Quantitation Kit (Thermo Fisher Scientific) and pooled in an equimolar manner (30 pM). A template was prepared with 510TM &520TM &530TM kit - Chef using the Ion Chef System. Up to 32 samples were loaded on Ion 530 chips (Thermo Fisher Scientific). The number of samples per chip was calculated to obtain an average cover of at least 450/500x. Data were analyzed with Ion Reporter Software v5.18 and the following stringent criteria were applied for final variant calling: (1) Class 4 or 5 variants, according to ACMG and ClinVar classifications; (2) allele frequency ≥ 5%; (3) *P*-value (QC) ≤ 0.01.

### APC gene target sequencing

DNA extracted from snap frozen FAP biopsies was sequenced by Sanger sequencing to confirm the germline *APC* variant diagnosed in the sampled tissues. For each sample, the *APC* amplicon carrying the germline mutation was sequenced, based on the patients’ clinical reports. In detail, 30 ng of genomic DNA, extracted from fresh FAP biopsies, were amplified with AmpliTaq Gold DNA Polymerase (Life Technologies) with ProFlex™ 3 × 32-well PCR System (Thermo Fisher Scientific, Waltham, MA, USA). Sequencing was performed on PCR products purified with GeneJET PCR Purification Kit (Thermo Fisher Scientific). Primer sequences and annealing temperatures are reported in Supplementary Table 1.

### Microbial DNA extraction and 16 S rRNA amplicon sequencing

Microbial DNA was extracted using the DNeasy Blood and Tissue kit (Qiagen) as previously described [19]. Raw sequences from faecal and oral samples were processed using a pipeline combining PANDASeq [20] and QIIME 2 [21]. After filtering for length and quality, reads were binned into amplicon sequence variants (ASVs) using DADA2 [22]. Taxonomic assignment was carried out on the SILVA database (v. 138.1) [23].

More details are described in the Supplementary Methods.

### Statistical analysis

qPCR and IHC data were analysed using GraphPad Prism 9.0. Data distribution was checked by the Shapiro-Wilk test. Ordinary one-way ANOVA with Tukey’s multiple comparison test or Kruskal-Wallis with Dunn’s multiple comparison tests were performed to assess significance. For microbiota, all statistical analyses were performed using R (v. 4.3.0). The ggplot2 and ternary (10.5281/zenodo.1068996) packages were used to generate Principal Coordinates Analysis (PCoA) and ternary plots, respectively. Data separation in the PCoA was tested using the pairwise.adonis package in R, which uses a permutation test with pseudo-F ratios (PERMANOVA). To examine between-group variation in alpha diversity and microbial composition at different taxonomic levels, the Kruskal-Wallis test was applied, followed by post-hoc Wilcoxon tests. Correlations between variables were computed using Spearman’s rank correlation coefficient, with the R cor.test function and the ggplot2 package used to generate correlation plots. Statistical significance was assessed as * *P* < .05, ** *P* < .01, *** *P* < .001, **** *P* < .0001. A *P*-value < 0.1 was considered a trend.

## Results

### Genetic characterization of tissue samples

Sanger sequencing analysis of FAP-tissues (NM and P) confirmed the inherited germline *APC* mutations in all samples except for FAP 06 P, probably due to a sensitivity issue. The germline *APC* mutations were frameshift (47%), nonsense (29%) and splicing variants (24%) (Supplementary Table 2). Through NGS analysis we found thirteen somatic mutations in twelve out of fifteen adenomas, among which ten occurring in exon 16, two in exon 4 and one in exon 7. In addition, a *KRAS* missense mutation (c.38G > A) was found in FAP 13 P (Supplementary Table 2).

*APC* somatic mutations were also found in three FAP NM tissues, indicating a possible aberrant crypt focus. Interestingly, a missense mutation in *NOTCH1* (c.4742 C > T) with an allelic frequency of 13% and described as a variance of uncertain significance (VUS) on ClinVar database was found in FAP 20 NM (Supplementary Table 2).

Sporadic CRCs tumour stages were I (13%), II (40%) and III (47%) (Supplementary Table 3), distributed as follows: right (33%), descending (20%), sigmoid colon (27%), and rectum (20%). The most common mutations in CRC tissues were *APC* (60%), *TP53* (47%), *KRAS* (27%), *PIK3CA* (26%), *BRAF* (20%), *MSH3* (13%) and *GNAS* (13%). In the *APC* gene frameshift (64%) and nonsense (36%) mutations were found in exons 16 (82%), 8 (9%) and 9 (9%). The missense pathogenic mutations c.534G > A (*TP53*), c.35G > A (*KRAS*), c.1799T > A (*BRAF*) and c.1633G > A (*PIK3CA*) were the most frequent (Supplementary Table 3). DNA MMR deficiency and MSI were found in three CRC cases.

### Wnt-driven carcinogenesis

**Fig. 1:**
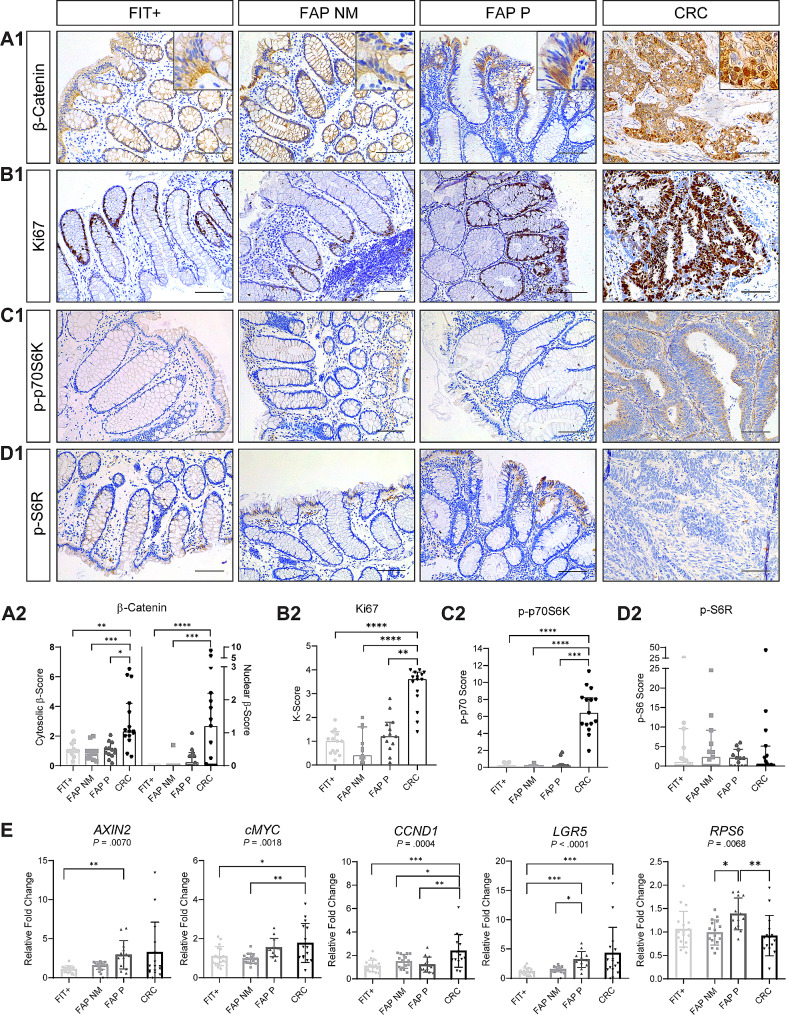
Transcriptional and translational signatures of Wnt/β-catenin and PI3K/mTOR in FIT+, FAP and CRC tissues. A1-D2) Representative images and relative quantifications of cytosolic and nuclear β-catenin (A1-A2), nuclear Ki67 (B1-B2), cytosolic p-p70S6K (C1-C2) and p-S6R (D1-D2); representative fields with a staining value close to the median of the corresponding group were shown for each marker. Values are shown as median with 95% CI, scale bars: 100 μm, magnification: 200x, magnification in small panels (A1) was three times higher that shown in the corresponding figures. Ten independent fields were quantified for CRC tissues while six to eight for FIT + and FAP tissues; *n* = 14 FIT+, 13 FAP NM, 13 FAP P, 15 CRC. E) RNA expression levels of *AXIN2, cMYC, CCND1, LGR5* and *RPS6* genes. Values are shown as mean ± SD; *n* = 17 FIT+, 16 FAP NM, 13 FAP P, 15 CRC. Ordinary one-way ANOVA with Tukey’s multiple comparison or Kruskal-Wallis with Dunn’s multiple comparison was performed accordingly to the data distribution checked with the Shapiro-Wilk test; * *P* < .05, ** *P* < .01, *** *P* < .001, **** *P* < .0001

The activation status of Wnt/β-catenin in FAP and CRC tissues was evaluated at both transcriptional and translational levels. Despite the presence of single-hit *APC* germline mutations, we found that subcellular localization of β-catenin protein in FAP NM was similar to FIT+, and mainly expressed in the cell membrane with no β-catenin nuclear translocation detected (Table 1; Fig. 1A1-A2). In contrast, β-catenin showed increased accumulation in the cytosol of FAP P (relative frequency distribution-RFD, medium + high: 54% FAP P vs. 29% FIT+), leading to its nuclear translocation (RFD, low + medium: 54% FAP P vs. 0% in FIT+) (Table 1; Fig. 1A1-A2).

**Table 1:** Median values of the staining scores and relative frequency distribution (RFD) of β-catenin (cytosolic and nuclear) staining, Ki67 nuclear staining, p-S6R and p-p70S6K cytosolic staining

			Relative frequency distribution (%)			
**Cytosolic β-catenin**	**Median**	**p value (vs. CRC)**	**Negative**	**Low**	**Medium**	**High**
FIT+	1	0.0020	21.4	50.0	21.4	7.1
FAP NM	0.8	0.0004	30.8	53.8	15.4	0
FAP P	1.1	0.0134	15.4	30.8	46.2	7.7
CRC	2.3		0	13.3	20.0	66.7
**Nuclear β-catenin**	**Median**	**p value (vs. CRC)**	**Negative**	**Low**	**Medium**	**High**
FIT+	0	< 0.0001	100	0	0	0
FAP NM	0	0.0009	92.3	0	0	7.7
FAP P	0.1	0.4416	46.2	46.2	7.7	0
CRC	1.2		26.7	13.3	6.7	53.3
**Ki67**	**Median**	**p value (vs. CRC)**	**Negative**	**Low**	**Medium**	**High**
FIT+	1	< 0.0001	28.6	28.6	42.9	0
FAP NM	0.4	< 0.0001	53.8	23.1	23.1	0
FAP P	1.2	0.0039	15.4	23.1	46.2	15.4
CRC	3.6		0	0	13.3	86.7
p-S6R	**Median**	**p value (vs. CRC)**	**Negative**	**Low**	**Medium**	**High**
FIT+	1	n.s.	42.9	28.6	0.0	28.6
FAP NM	2.3	n.s.	38.5	7.7	23.1	30.8
FAP P	2.1	n.s.	38.5	7.7	30.8	23.1
CRC	0.6		46.7	13.3	13.3	26.7
p-p70S6K	**Median**	**p value (vs. CRC)**	**Negative**	**Low**	**Medium**	**High**
FIT+	0	< 0.0001	85.7	14.3	0	0
FAP NM	0	< 0.0001	100.0	0	0	0
FAP P	0.2	0.0003	84.6	0	15.4	0
CRC	6.4		0	0	6.7	93.3

As expected, CRC tissues showed the highest accumulation of β-catenin in both the cytosol (RFD-high: 66.7%) and the nucleus (RFD-high: 53.3%) compared to FIT + and FAP tissues (Table 1; Fig. 1A1-A2). By qPCR we confirmed the stepwise activation of the Wnt signalling through the activation of target genes. Indeed, we found increased *AXIN2* expression from FAP NM (fold: 1.6) to FAP P (fold: 2.9) (*P* = .0042) and CRC tissues (fold: 3.3), and increased *cMYC* expression in FAP P (fold: 1.5) and CRC tissues (fold: 1.8) (*P* = .0154) compared to FIT+ (Fig. 1E). In contrast, *CCND1* levels increased significantly only in CRC tissues (fold: 2.4) (*P* = .0004) (Fig. 1E). Importantly, nuclear β-catenin was found to correlate positively with *cMYC*, *AXIN2* and *CCND1* expression (Spearman rho: 0.79, 0.65, 0.6; *P* ≤ .049) in FAP P and only with *AXIN2* in CRCs (Spearman rho: 0.57; *P* = .027) (Supplementary Fig. 2A-B), despite the higher accumulation of the protein in the nucleus.

The increased activation of Wnt signalling from FAP P to CRCs was confirmed also through the analysis of the intestinal stem cells marker *LGR5* and proliferation (Ki67 marker).

*LGR5* transcriptional levels increased stepwise from FIT+ (fold:1.2) to FAP P (fold: 3.2) (*P* = .0001) and CRC tissues (fold:4.3) (*P* = .0007) (Fig. 1E). Accordingly, 87% of CRCs had a higher expression of Ki67 compared to FAP P (15%) (*P* = .0039) and FIT + tissues (0%) (*P* < .0001) (Table 1; Fig. 1B1-B2). In FAP NM tissues Ki67 expression slightly decreased compared to FIT+ (fold: 0.4) while *LGR5* expression slightly increased (fold: 1.5) but appeared significantly lower than the adenoma group (*P* = .019). Results are further described in the Supplementary Material.

### PI3K/mTOR deregulation in APC-mutated colorectal cancer

P-p70S6K protein levels were found to be significantly higher in CRCs compared to FIT + and FAP tissues (*P* < .0001) (Fig. 1C1-C2; Table 1). In fact, 100% of CRC tissues showed medium-high expression of p-p70S6K compared to FAP P (15%), FAP NM (0%) and FIT+ (both 0%). In the same tissues the expression of *RPS6* gene (fold: 0.9) and p-S6R protein (median: 0.6) did not change significantly (Fig. 1D1-D2, 1E; Table 1). However, a negative correlation between p-S6R protein and *RPS6* RNA expression was found in CRCs carrying mutations directly and indirectly involved in the mTOR pathway (*PIK3CA* and/or *KRAS*) (Supplementary Fig. 2C). Despite the lower activation of p-p70S6K, in FAP P we found an overexpression of *RPS6* gene (fold:1.4) and p-S6R protein (median: 2.1) compared to FIT + and CRC tissues (Fig. 1D1-D2 and 1E; Table 1). Similar results were found in FAP NM (p-S6R protein expression median: 2.3) compared to FIT + and CRC tissues, but without changes in *RPS6* expression (Fig. 1D1-D2 and 1E; Table 1). Furthermore, a negative correlation between p-S6R protein and *RPS6* gene was found in FAP NM tissues (rho: -0.6; *P*: 0.051) (Supplementary Fig. 2D), together with a positive correlation between p-S6R protein and the cytosolic β-catenin (rho: 0.74; *P* = .004) (Supplementary Fig. 2D).

### Characterization of oral, faecal and mucosa-associated microbiota

**Fig. 2:**
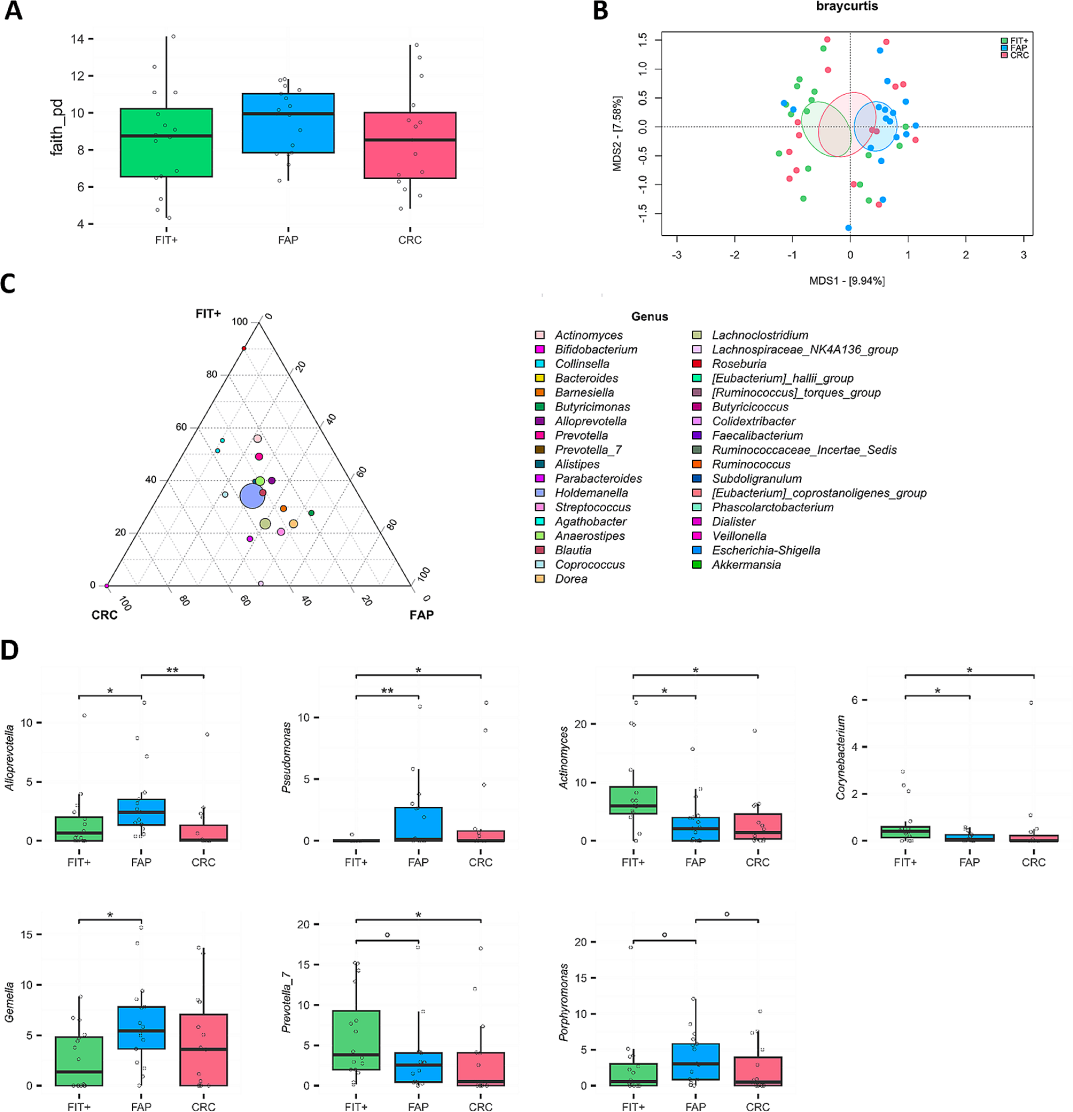
Oral microbiota in FAP and CRC patients compared to FIT + subjects. (**A**) Distribution of alpha diversity, calculated using Faith’s phylogenetic diversity, in the oral microbial profiles of FAP, CRC and FIT + subjects. No significant differences were found (Wilcoxon test, *P* > .05). (**B**) PCoA of Bray-Curtis dissimilarity between microbial profiles. Ellipses include a 95% confidence area based on the standard error of the weighted average of sample coordinates. A significant separation was found between the FAP and FIT + groups (Adonis, *P* = .0084). (**C**) Ternary plot showing the genus-level composition of the three groups, with point size representing the mean relative abundance in the cohort. The position of each point indicates which group is more represented by that taxon. (**D**) Relative abundance distribution of bacterial genera differentially represented between groups. Wilcoxon test, ° *P* < .1; * *P* < .05; ** *P* < .01. *n* = 16 FIT+, 17 FAP and 15 CRC.

The oral microbiota of FAP patients segregated significantly from that of the FIT + group in the PCoA of Bray-Curtis dissimilarity (*P* = .0084), but no differences were found in alpha diversity (Fig. 2A and B). At the genus level, FAP patients were particularly enriched in *Pseudomonas*, *Gemella*, *Alloprevotella* and *Porphyromonas* (*P* ≤ .1), while FIT + patients were enriched in *Actinomyces*, *Corynebacterium* and *Prevotella_7* (*P* ≤ .084) (Fig. 2C − 2D).

**Fig. 3:**
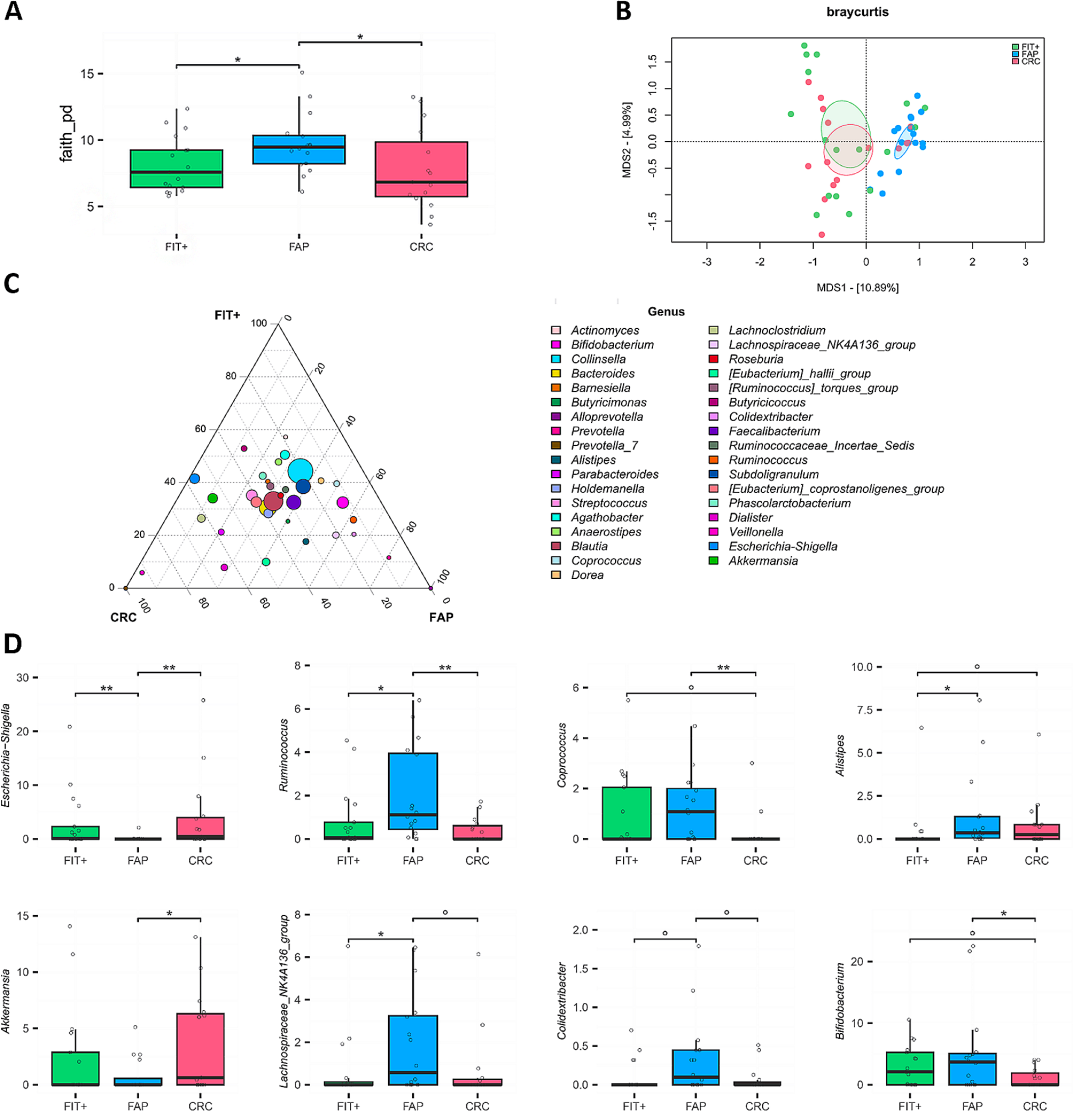
Faecal microbiota in FAP and CRC patients compared to FIT + subjects. (**A**) Distribution of alpha diversity, calculated using Faith’s phylogenetic diversity, in the faecal microbial profiles of FAP, CRC and FIT + subjects. Wilcoxon test, * *P* < .05. (**B**) PCoA of Bray-Curtis dissimilarity between microbial profiles. Ellipses include a 95% confidence area based on the standard error of the weighted average of sample coordinates. A significant separation was found between FAP and the other groups (Adonis, *P* = .0005). (**C**) Ternary plot showing the genus-level composition of the three groups, with point size representing the mean relative abundance in the cohort. The position of each point indicates which group is more represented by that taxon. (**D**) Relative abundance distribution of bacterial genera differentially represented between groups. Wilcoxon test, ° *P* < .1; * *P* < .05; ** *P* < .01. *n* = 17 FIT+, 16 FAP and 15 CRC.

Looking at the faecal microbiota, FAP patients showed higher alpha diversity compared to both the CRC and FIT + groups (*P ≤* .049) and significantly segregated from them in the Bray-Curtis PCoA (*P* ≤ .0005) (Fig. 3A and B). At the genus level, CRC patients were enriched in *Escherichia-Shigella* and *Akkermansia* compared to FAP patients (*P* ≤ .023), who showed higher relative abundances of *Ruminococcus*, *Coprococcus*, *Alistipes*, *Lachnospiraceae*_NK4A136_group, *Colidextribacter* and *Bifidobacterium* (*P* ≤ .082) (Fig. 3C and D).

**Fig. 4:**
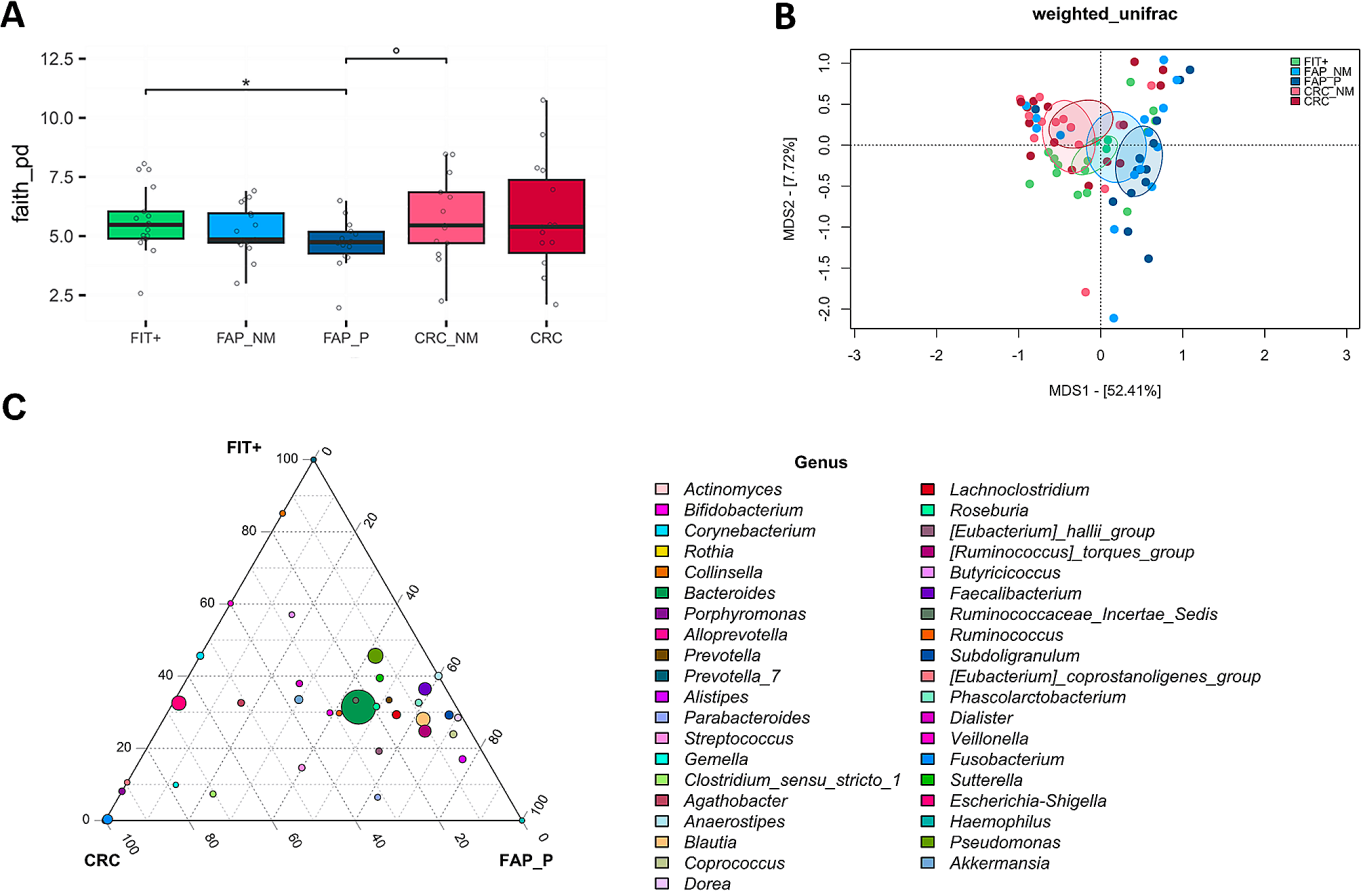
Mucosa-associated microbiota in FAP and CRC patients compared to FIT + subjects. (**A**) Distribution of alpha diversity, calculated using Faith’s phylogenetic diversity, in the microbial profiles associated with adenomatous polyps from FAP patients (FAP P), cancerous mucosa from CRC patients (CRC) and normal mucosa from FAP (FAP NM), CRC (CRC NM) and FIT + subjects (FIT+). Wilcoxon test, ° *P* < .1; * *P* < .05. (**B**) PCoA of Bray-Curtis dissimilarity between microbial profiles. Ellipses include a 95% confidence area based on the standard error of the weighted average of sample coordinates. A significant separation was found between all groups (Adonis, *P* < .05). (**C**) Ternary plot showing the genus-level composition of the three groups, with point size representing the mean relative abundance in the cohort. The position of each point indicates which group is more represented by that taxon. Wilcoxon test, ° *P* < .1; * *P* < .05; ** *P* < .01; *** *P* < .001. *n* = 17 FIT+, FAP NM, 14 FAP P, 13 CRC NM and 15 CRC.

Regarding the mucosa-associated microbiota, higher alpha diversity was found in the FIT + group compared to the FAP P group (*P* = .029), and a significant segregation between all groups was observed in the Bray-Curtis PCoA (*P* < .05) (Fig. 4A and B). At the genus level (Fig. 4C and Supplementary Fig. 6E), CRC tissues were enriched in *Corynebacterium*, with CRC being particularly characterized by increased proportions of *Fusobacterium* and *Escherichia-Shigella* (*P* < .05). However, no significant differences emerged in beta diversity among CRC tissues with different TNM stage-based categories (Supplementary Fig. 10A). The FAP P group was mainly discriminated by an overabundance of *Ruminococcaceae* and *Lachnospiraceae* taxa, such as *[Ruminococcus]_torques_group*, *Subdoligranulum*, *Phascolarctobacterium*, *Faecalibacterium*, and *Roseburia*, along with *Alistipes* (*P* < .05).

Importantly, looking at age-driven microbiome differences, we found a significant increase in the relative abundance of the *Subdoligranulum* genus (*P* = .002) in the faecal composition of the FAP group (median age < 28 years) (Supplementary Fig. 8D) and an enrichment in *Faecalibacterium* (*P* = .02) in the mucosal-associated microbiome of FIT + subjects under 65 years (Supplementary Fig. 9F). More details are described in the Supplementary Results.

### Microbiota-Wnt/β-catenin and PIK3CA/mTOR correlations in FAP and CRC tissues

**Fig. 5:**
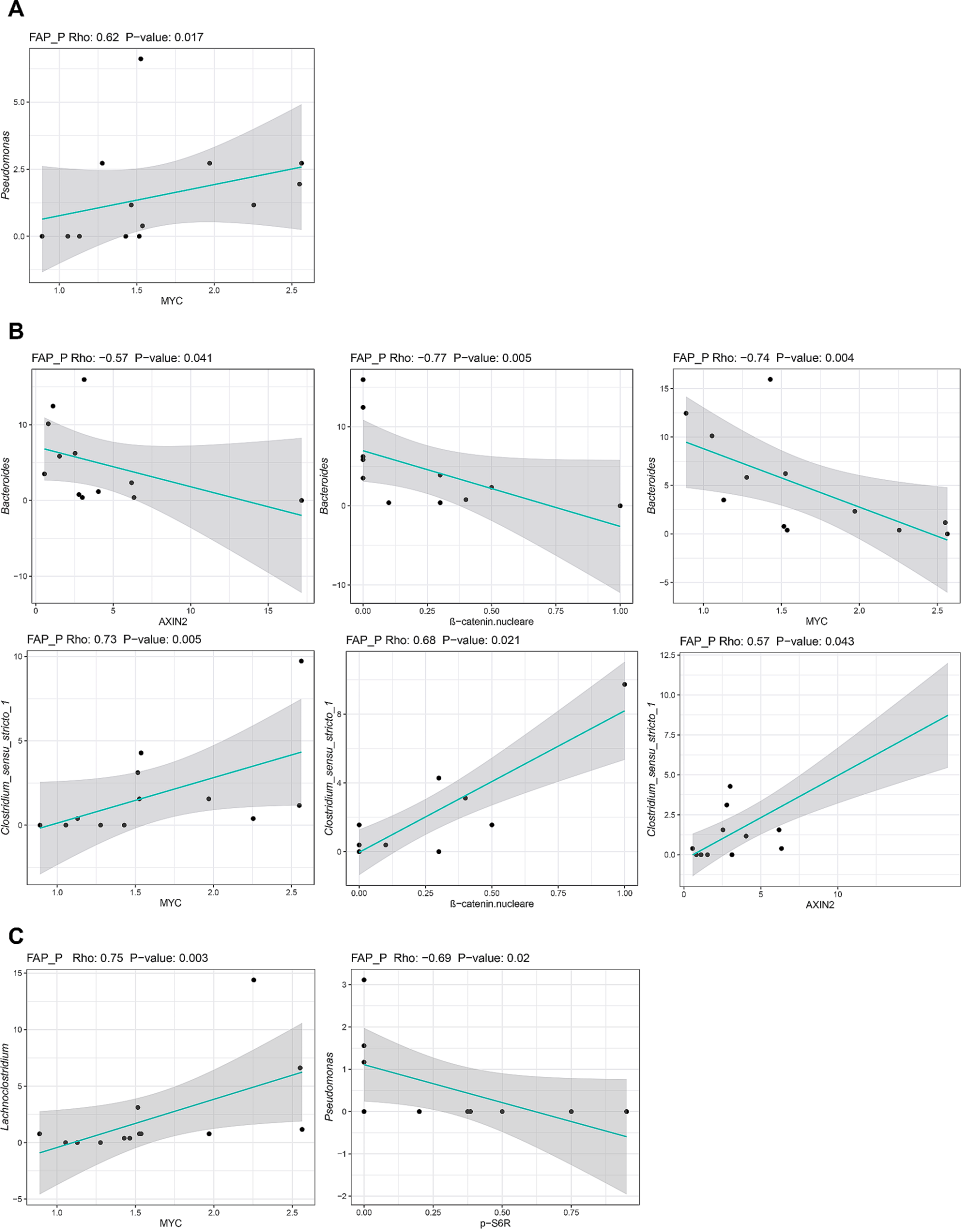
Associations between Wnt/β-catenin and mTOR downstream effectors and relative abundances of oral, faecal and mucosa-associated bacterial genera in FAP patients. Correlation plots between RNA (*i.e. MYC*, *AXIN2* and *CCND1*) or protein (i.e. nuclear β-Catenin and p-S6R) expression levels and relative taxon abundances in the oral (**A**), faecal (**B**) and mucosal (**C**) microbiota of FAP patients. Only significant Spearman’s correlations (*P* < .05) at family and genus level with |rho| > 0.3 are shown

Relevant molecular markers associated with FAP P or CRC groups were correlated with the relative abundance of bacterial genera in the oral, faecal and mucosa-associated microbiota. We found that oral *Pseudomonas* correlated positively with *cMYC* expression (rho: 0.62; *P* = .017) in FAP P (Fig. 5A). At the faecal level, *Bacteroides* correlated negatively with FAP P nuclear β-catenin, *AXIN2* and *cMYC* expression (rho: -0.77, -0.57, -0.74; *P* = .005, 0.041, 0.004), while *Clostridium_sensu_stricto_1* showed a positive correlation with nuclear β-catenin, *AXIN2* and *cMYC* (rho: 0.68, 0.57, 0.73; *P* = .021, 0.043, 0.005) (Fig. 5B). Furthermore, FAP P mucosa-associated *Lachnoclostridium* positively correlated with c*MYC* expression (rho: 0.75; *P* = .003) and *Pseudomonas* correlated negatively with p-S6R (rho: -0.69; *P* = .02) (Fig. 5C).

**Fig. 6:**
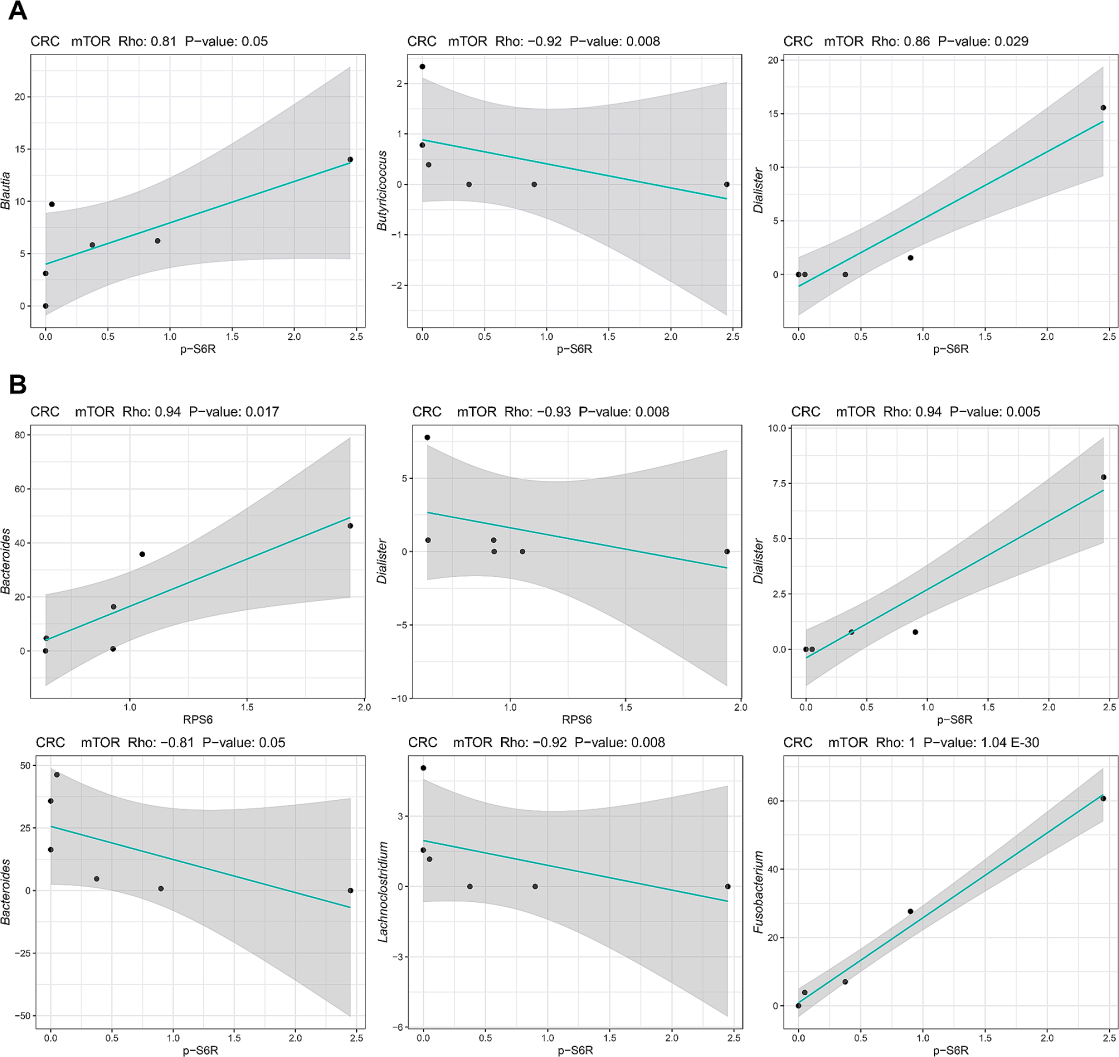
Associations between mTOR downstream effectors and relative abundances of faecal and mucosa-associated bacterial genera in CRC patients. Correlation plots between RNA (i.e. *RPS6*) or protein (i.e. p-S6R) expression levels and relative taxon abundances in the faecal (**A**) and mucosal (**B**) microbiota of CRC patients. Only significant Spearman’s correlations (*P* < .05) at family and genus level with |rho| > 0.3 are shown

In CRCs samples carrying mutations in the PI3K/mTOR pathway, the expression of p-S6R correlated positively with faecal *Dialister* (rho: 0.86; *P* = .029) and *Blautia* (rho: 0.81; *P* = .05) and negatively with faecal *Butyricicoccus* (rho: -0.92; *P* = .008) (Fig. 6A). In the same tissues, mucosa-associated *Bacteroides* and *Dialister* correlated positively and negatively with *RPS6* gene expression respectively (rho: 0.9, -0.93; *P* ≤ .017); *Dialister* and *Fusobacterium* showed a positive correlation with p-S6R expression (rho: 0.94, 1; *P* ≤ .005), whereas *Bacteroides* and *Lachnoclostridium* showed a negative correlation (rho: -0.81, -0.92; *P* ≤ .05) (Fig. 6B). Interestingly, *Bacteroides* also correlated positively with the expression of *AXIN2* (rho: 0.97; *P* = .001) and *cMYC* (rho: 0.97; *P* = .001) gene expression in CRCs samples with a low immunoscore (IS: I0-I1-I2) (Supplementary Fig. 11C). More details are described in the Supplementary Results.

## Discussion

This study compares the genetic, molecular and microbiota profile of healthy (FIT+), FAP and CRC patients. We found several distinctive signatures of the three cohorts that correlated with each other and among patients, which represent different levels of Wnt derangement given either by somatic or germline mutation in *APC*.

In our cohort, FAP NM tissues, carrying a mutation in one copy of the *APC* gene, differed from healthy FIT + NM by enhanced Wnt/β-catenin and PI3K/mTOR activation, and are characterized by a higher expression of *AXIN2*, *CCND1*, *LGR5* Wnt target genes and the mTOR downstream effector protein p-S6R, as previously reported in different models such as the *Apc*^Δ716^ heterozygous mutant mouse [24]. The abundance of cytosolic β-catenin was found to positively correlate with p-S6R expression specifically in these tissues, supporting the relevance of the WNT-mTOR interplay in *APC*-deranged carcinogenesis at very early stages. Furthermore, a distinctive mucosa-associated microbiota with lower proportions of the Actinobacteriota, Patescibacteria and Acidobacteriota phyla was found in FAP NM compared to FIT+, suggesting an already impaired microbiota-epithelial dialogue.

The occurrence of somatic *APC* gene mutations induces a more severe Wnt signalling derangement in FAP P compared to FAP NM, with increased cytosolic and nuclear β-catenin, which positively correlated with *AXIN2, CCND1* and *cMYC* gene expressions [25, 26]. A higher p-S6R expression was found also in FAP P compared to FIT + and CRC tissues, although no significant correlations were found with cytosolic or nuclear β-catenin expression, as for FAP NM. These results support the use of mTOR inhibitors, such as Rapamycin, as selective treatment against CRC development and progression in *APC*-mutated settings [27].

Interestingly, FAP P can be distinguished from FAP and FIT + NM tissues by a positive correlation between the Wnt/β-catenin molecular markers, nuclear β-catenin, *AXIN2* and *cMYC*, and FAP-faecal *Clostridium_sensu_stricto_1*, which is one of the main intestinal genera reported in the literature to be positively associated with CRC [28, 29]. In addition, the same FAP P Wnt/β-catenin molecular markers negatively correlated with FAP-faecal *Bacteroides*, suggesting that the faecal microbiota composition may predict the Wnt/β-catenin activation status of the tissue in FAP patients.

Moreover, an enrichment in typically dominant gut taxa (such as *Lachnospiraceae* and *Ruminococcaceae*), but also in the [*Ruminococcus*] *torques* group and *Alistipes* was found in FAP P-faecal and mucosal associated microbiota. It should be noted that [*Ruminococcus*] *torques* is a pro-inflammatory mucolytic taxon associated with a higher risk of developing CRC [30, 31], similar to *Alistipes*, a bile-resistant genus that may promote CRC via the IL-6/STAT 3 pathway [32].

FAP faecal microbiota was also enriched of *Bifidobacterium* and *Colidextribacter*. The latter has been suggested to be involved in the development of CRC and to interfere with the efficacy of immune checkpoint inhibitors [33, 34].

FAP tissues (P and NM) showed an overabundance of *Lachnoclostridium* compared to CRC NM and cancer tissues, and showed a positive correlation with *cMYC* expression in FAP P.

These data are particularly important, as the use of *Lachnoclostridium* has been proposed as a non-invasive bacterial marker with FIT to improve the diagnostic sensitivity of the screening test, as it showed higher sensitivity to colorectal adenomas (non-advanced and advanced) [35] than *Fusobacterium* [36]. Since the latter appeared to be more specific for advanced CRC [36], our results support the possibility to find adenoma-specific signatures to effectively detect precancerous lesions.

In terms of oral microbiota, FAP patients differentiated by higher proportions of *Alloprevotella, Pseudomonas* and *Gemella*. Both *Alloprevotella* and *Gemella* were found to be significantly high in CRC [37, 38], strengthening the predictive value of oral microbiota for CRC screening. Importantly, *Pseudomonas* correlated with *cMYC* and p-S6R in the oral and mucosa-associated ecosystem, respectively, supporting the relevance of oral microbiota profiling in CRC early detection.

In contrast to FAP tissues, CRCs showed hyperactivation of the Wnt pathway which may interfere with the activation of mTOR signalling since the expression of the downstream effector p-S6R protein did not change. Indeed, in mTOR-mutated CRCs (*PIK3CA* and/or *KRAS* gene mutations), the negative correlation trend between *RPS6* RNA and p-S6R protein expression suggests the involvement of post-transcriptional regulatory mechanisms.

Furthermore, p-S6R correlated with the faecal genera *Blautia* and *Butyricicoccus*, which may serve as additional biomarkers to improve the efficacy of CRC screening. More importantly, p-S6R protein levels positively correlated with *Dialister* relative abundance in the mucosal ecosystem. Interestingly, the same correlation was also found in the faecal ones. Since *Dialister* has been associated with higher cytotoxicity in irinotecan-treated CRC patients [39], our data suggest that the use of this strain, as faecal biomarker, may potentially predict the toxicity of chemotherapy in CRCs with impaired mTOR signalling pathway.

As expected, we found a higher abundance of *Fusobacterium* in CRCs compared to the FAP and FIT + groups, which positively correlated with the p-S6R protein in mTOR-mutated CRCs. Notably, a direct correlation between *F. nucleatum*, active ERBB2-PIK3-AKT-mTOR pathway and a higher tumor mutation burden has been previously described in gastric cancer patients [40]. In addition, an enrichment in polyps- and tumors-associated taxa such as *Corynebacterium* and *Escherichia-Shigella* was found in the CRC mucosal-microbiota [41, 42]. Particularly, *Corynebacterium appendicis* has recently been included in a panel of tumor tissue-specific bacterial biomarkers for CRC [43]. Interestingly, increased relative abundance of *Bacteroides* positively correlated with *AXIN2* and *cMYC* gene expression in CRCs with low immunoscore. Indeed, the *B. fragilis* zinc-dependent metalloprotease toxin was found to interact with E-cadherin, leading to the disruption of intercellular junctions, and promoting the activation of Wnt signaling-associated proto-oncogenes, such as *cMYC* [44, 45]. Several further mechanisms by which members of *Bacteroides* can act as opportunistic pathogens for the host gut have been described [46]. Particularly, the ability to induce mucus layer degradation [47] or modulate IL-10 by the *B. fragilis* polysaccharide A (PSA) [46] can be responsible for an immunosuppressed microenvironment that favours tumour progression. A direct relationship between TLR activation, tumour-derived WNT ligands and TLR-activated monocytes secreting IL-10 has been previously described in lung cancers [48]. Since low immunoscore is associated with poor overall survival and disease free survival in patients with CRC [49, 50], our data may support a role of *Bacteroides* as potential predictive biomarker for CRC prognosis.

An overabundance of *Escherichia-Shigella* along with *Akkermansia* also characterized the CRC faecal microbiota. It has been shown that *Akkermansia* promotes CRC possibly by exacerbating early-stage inflammation and increasing intestinal epithelial cell proliferation [51, 52]. Furthermore, the oral microbiota of our cohort of CRC patients showed a reduction in the commensal taxa *Actinomyces*, *Corynebacterium*, and *Prevotella_7*, confirming a dysbiosis also in the oral niche.

Due to the complexity and heterogeneity of CRC together with the very stringent inclusion criteria, the main limitations of the study include the small number of eligible patients and the wide age range, including older adults in the CRC cohort. Indeed, age is a major driver of microbiota variation [53]. As described in the supplementary material, we stratified FIT + and CRC subjects into adults and elderly using 65 years as the threshold, and FAP patients (the youngest group in our cohort) by the median age of 28 years. This analysis only found the abundance of *Faecalibacterium* taxon in the mucosal samples as a possible limited marker for FAP, while most of the above microbial signatures were consistent with findings available in the literature, supporting that data were driven by limited sample size- and age-related bias. In addition, we cannot rule out a potential confounding effect due to the parentage of most of the FAP patients, although diet and environmental habits mostly influence the composition of the microbiota [54]. Tumour stage and the site of CRC sampling could also be another limitation of the study. Indeed, by stratifying CRC tissue samples into two groups based on the pathological pT stages, identified as “Low T” (pT1 and pT2) and “High T” (pT3), we found no significant differences in the mucosal microbiota composition.

To the best of our knowledge, robust validation cohorts and datasets correlating molecular and microbiota profiling of FAP and CRC patients are not yet available in the literature. The strength of our study is related to the specific correlation panel of genetic, molecular and microbiota features of the three clinical groups representing the multistage progression of CRC. Particularly, the identification in FAP patients of a specific microbiota composition with some taxa found both in mucosal and faecal specimens and associated with the specific derangement of Wnt/β-catenin and PI3K/mTOR signallings may help in developing assays to differentiate patients with early-stage adenomas often missed by FIT screening.

Further investigations are required to validate our results in larger cohorts, however this study aims at helping to set some criteria for the use of microbiota in association with conventional screening programs for the early detection of premalignant lesions in both sporadic and hereditary settings. In addition, the close relationship between molecular and microbiota profiling, highlighted in our study, suggests that multitarget biomarkers and therapies should be considered for successful CRC prevention, early detection, and treatment.

### Electronic supplementary material

Below is the link to the electronic supplementary material.

Supplementary Material 1

## Data Availability

The authors declare that the data supporting the findings of this study are available within the paper and its supplemental information files.

## Abbreviations

APC: Adenomatous polyposis coli
CRC: Colorectal cancer
FAP: Familial adenomatous polyposis
FIT: Faecal immunochemical test
mTOR: Mammalian target of rapamycin
NM: Normal appearing colonic mucosa
P: Polyp/adenoma
