# Supplementary material for: Interplay between WNT/PI3K-mTOR axis and the microbiota in APC-driven colorectal carcinogenesis: data from a pilot study and possible implications for CRC prevention
Source: J Transl Med. 2024 Jul 5;22:631. doi: 10.1186/s12967-024-05305-5 (PMC11227240; doi:10.1186/s12967-024-05305-5)
Supplement: Supplementary file 1 — Supplementary Material 1 [file 12967_2024_5305_MOESM1_ESM.docx]

**Supplementary material**

**METHODS**

**Patient characteristics**

In our FAP cohort, 12 patients shared family history in pair, while FAP04 and FAP09 had the diagnosis of attenuated FAP (AFAP) and no adenomas were found during surveillance colonoscopy. Thus, 15 patients with adenomas out of 17 FAP patients enrolled were available for the analysis. The geographic background of the enrolled patients was European except for a CRC patient who was Caucasian.

Patients with a history of inflammatory bowel disease, hereditary gastrointestinal syndromes other than FAP, severe co-morbidities with reduced life expectancy, those with inoperable metastatic CRC or with a personal history of CRC, and those who had used antibiotics/probiotics in the three months prior to enrolment were excluded from the study. The exclusion criteria were introduced in order to analyse subsets of homogeneous populations and prevent genetic or microbiota-related bias.

**Immunohistochemical analysis**

*Relative frequency distribution*

The calculation of the relative frequency distribution required several steps.

First, raw data of each case/group (positive/total nuclei (%) for Ki67 and IS score (0-12) for β-catenin, p-p70S6K and pS6R) were normalized to the median of the raw data of FIT+ cases respectively to obtain the staining score. Thereafter, four frequency classes (negative – low – medium - high) were created for each marker. The number of samples with a staining score within the range of each frequency class was indicated (we referred to this data distribution as Absolute Frequency Distribution). Subsequently, the relative frequency distribution (%) was calculated by dividing the absolute frequency distribution of each frequency class by the total number of samples (n) within each patient group. The final value was multiplied by 100.

Below, we provide an example of the entire analysis process for Ki67 marker.

1. Raw data in % (mean percentage of the ratio between positive nuclei and total number of nuclei per crypt calculated for a total number of 6-10 crypts/sample are shown for each case/group (Supplementary Table 5A).
2. The single values of each group were normalized to the median of the raw data of FIT+ cases (for Ki67 value: 24,84) to obtain the Ki67Score/sample (K-Score) (Supplementary Table 5B). The K-Scores of each patient group are represented in Figure 1 B2 and their median values are shown on Table 1.
3. The K-Scores of each case/group (FIT+, FAP NM, FAP P, CRC NM, CRC) were grouped into four frequency classes with the following staining score ranges:

0 – 0.5 = Negative

0.5 – 1 = Low

1 – 2 = Medium

>2 = High

The number of samples with a K-Score within each frequency class range was indicated. This value represents the Absolute Frequency (Supplementary Table 5C).

1. Finally, the Relative Frequency Distribution (%) was calculated as the ratio between the Absolute Frequency and the number of samples in each group (n) multiplied by 100 (Supplementary Table 5C).

The relative frequency distribution values (%) for each marker are shown in Table 1. We used the calculation of the Relative Frequency Distribution of Ki67 as an example of the calculation method, however, the calculation for the other markers was performed similarly.

*Determination of the Immunoscore*

Formalin-fixed paraffin-embedded tumour samples were incubated for 16 minutes at 36°C with rabbit monoclonal antibodies against CD8 (SP239; Ventana) and 16 minutes at room temperature with rabbit monoclonal antibodies against CD3 (2GV6; Ventana). The OptiView DAB IHC Detection Kit (Ventana) was applied for detecting primary antibodies. High-resolution digital slides at 20X were obtained with a Ventana DP200 slide scanner (Ventana). The density of stained cells in the tumour areas and invasion margins was determined using the computerized image analysis software QuPath v. 0.5.1 under the supervision of an experienced pathologist (CC) (1).

Each tumour was categorized into High or Low density for each marker in each tumour region, according to a predetermined cut-off value (CD3 CT, 647 cells/mm^2^; CD3 IM, 1343 cells/mm^2^; CD8 CT, 235 cells/mm^2^; CD8 IM, 541 cells/mm^2^) using the median value of all cases/marker/tumour region respectively (Supplementary Table 6).

Patients were stratified according to a score ranging from I0 to I4 depending on the total number of densities observed (two markers assessed in CT, two markers assessed in IM) (2–5). Finally, patients were divided into two IS groups based on their IS value, I0-I1-I2: Low and I3-I4: High to perform the correlation analysis with the microbiota.

**Microbial DNA Extraction Protocols**

Microbial DNA was extracted from oral swabs, mucosal biopsies and faeces using the DNeasy Blood and Tissue kit (Qiagen) with minor modifications according to the type of sample. Briefly, for oral samples, the biological material was detached from the swab by adding sterile physiological solution and then vortexing. The pellet was separated by centrifugation at 8,000 rpm for 10 min, resuspended in 180 μl of enzymatic lysis buffer (500 mM NaCl, 50 mM Tris-HCl pH 8, 50 mM EDTA, 4% sodium dodecyl sulfate, and 20 mg/ml lysozyme) and incubated at 37°C for 30 min. The samples were then processed following the DNeasy Blood and Tissue kit (QIAGEN) protocol as described for faeces and biopsies. For faecal samples, microbial DNA was extracted using the repeated bead-beating plus column protocol, as described in D’Amico et al. (6). Briefly, 250 mg of faecal samples were mixed with 1 ml of lysis buffer (500 mM NaCl, 50 mM Tris–HCl pH 8, 50 mM EDTA, 4% (w/v) SDS), along with four 3-mm glass beads and 0.5 g of 0.1-mm zirconia beads (BioSpec Products). The samples were homogenized using a FastPrep instrument (MP Biomedicals) with three bead-beating steps at 5.5 movements/sec for 1 min, and incubated on ice for 5 min between treatments. The stool particles were then pelleted by centrifugation at 13,000 rpm for 5 min. Nucleic acids were precipitated by adding 260 μl of 10 M ammonium acetate and one volume of isopropanol. The resulting pellets were washed with 70% ethanol and suspended in TE buffer. To remove RNA, 2 μl of DNase-free Rnase (10 mg/ml) was applied at 37 °C for 15 min. Protein removal and column-based DNA purification were performed following the DNeasy Blood and Tissue kit protocol (QIAGEN). For mucosal biopsies, tissue-adherent microbes were separated by vortexing in sterile physiological solution. Samples were centrifuged at 8,000 rpm for 5 min to remove tissues, and supernatants were processed similarly to the faecal samples, starting with the repeated bead-beating plus column protocol. For all samples, DNA quantification was performed using the NanoDrop ND-1000 spectrophotometer (NanoDrop Technologies). For all samples, libraries of the V3-V4 hypervariable region of the 16S rRNA gene were prepared following the “16S Metagenomic Sequencing Library Preparation” Illumina protocol. Sequencing was performed on an Illumina MiSeq platform using a 2 × 250 bp paired-end protocol according to the manufacturer’s instructions. Raw sequences of mucosal biopsies were first cleaned to remove any human contaminants using the Kneaddata tool (7). Next, the analysis workflow taxonomic assignment, using the VSEARCH algorithm (8) on the SILVA database (version 138.1, released 11 Nov 2020), also included the exclusion of chimeras.

Alpha diversity was calculated using several metrics, including the Shannon index, the number of observed features and Faith’s phylogenetic diversity. Beta diversity was computed using Bray-Curtis dissimilarity, and weighted and unweighted UniFrac distances, which were then used as input for Principal Coordinate Analysis (PCoA).

**RESULTS**

## **Stemness and proliferation features of tissues samples**

Activation of the Wnt/β-catenin pathway, above all at transcriptional levels, was also found in CRC NM tissues, as suggested by increasing trend levels of *AXIN2* (fold: 1.6)*, cMYC* (fold: 2.3; *P* = .0001)*, CCND1* (fold: 1.6) and cytosolic β-catenin (median: 1.3) compared to FIT+ (Supplementary Figures 1A and 1C). Furthermore, in CRC NM tissues we found higher *LGR5* (fold:1.8) and Ki67 (median:1.3) expression compared to FIT+. This suggests the presence of diffuse proliferative cells up to 5 cm from tumour localization in response to the spread of proliferative signals (Supplementary Figures 1A and 1D).

In the same tissues we found an overexpression of *RPS6* (fold RNA expression: 1.8, *P* = .0009 vs FIT+, *P* < .0001 vs CRC; median active protein expression: 7.6) and p-p70S6K (median: 1.7), highlighting the activation of the corresponding signalling pathway (Supplementary Figures 1B and 1E).

In addition, we investigated the expression of two important cancer-related genes, the vascular endothelial growth factor-A (*VEGF-A*) and tumour suppressor gene *TP53.* We found a slight increase in *VEGF-A* levels in CRC (fold: 2.1) and CRC NM (fold: 1.4) tissues compared to FIT+; this is in line with their higher proliferation rates (Supplementary Figure 1D).

Interestingly, CRC and FAP tissues showed no increased expression of *TP53* which increased significantly in CRC NM (fold: 1.8) (Supplementary Figure 1D).

**Oral, faecal and mucosa-associated microbiota**

The ecosystems differed markedly in alpha and beta diversity, with the faecal and mucosal samples being more similar to each other than the oral samples (Supplementary Figure 3). Nevertheless, several differences emerged between FAP, CRC and FIT+ subjects.

From a taxonomic point of view, the oral microbiota of all patients was mainly accounted for by the phylum Firmicutes (mean relative abundance in FIT+ vs. FAP vs. CRC, 47.8% vs. 44.7% vs. 47.7%), followed by Proteobacteria (12.3% vs. 23.1% vs. 18.8%), Bacteroidota (17.3% vs. 16.2% vs. 12.9%), Actinobacteriota (12.9% vs. 7.4% vs. 8.9%), and Fusobacteriota (5.9% vs. 5.4% vs. 5.8%) (Supplementary Figure 4A). At the family level, *Streptococcaceae* (27.4% vs. 24.7% vs. 28.1%), *Pasteurellaceae* (7.4% vs. 11.3% vs. 11.7%) and *Prevotellaceae* (12.3% vs. 10.3% vs. 7.3%) dominated, followed by *Veillonellaceae* (8.8% vs. 6.8% vs. 6.6%), *Neisseriaceae* (4.3% vs. 8.4% vs. 4.6%), and *Actinomycetaceae* (8.2% vs. 3.2% vs. 3.3%) (Supplementary Figure 4B). Interestingly, some signatures characterized FAP patients compared to the FIT+ group, including a higher representation of the phylum Proteobacteria (Wilcoxon test, *P* = .025) and the families *Pseudomonadaceae*, *Gemellaceae*, and *Lactobacillaceae* (*P* ≤ .04) (Supplementary Figure 4C). FAP patients also tended to be enriched in *Porphyromonadaceae* compared to the other groups (*P* ≤ .1). On the other hand, CRC patients showed higher proportions of *Pseudomonadaceae* compared to FIT+ subjects (*P* = .011), which were discriminated mainly by increased levels of *Actinomycetaceae*, *Corynebacteriaceae*, *Selenomonadaceae*, and *Peptostreptococcaceae* (*P* ≤ .044) (Supplementary Figure 4D).

The faecal microbial ecosystem of all subjects was dominated by the phylum Firmicutes (mean relative abundance in FIT+ vs. FAP vs. CRC, 68.7% vs. 68.4% vs. 66.6%), along with Actinobacteriota (17.1% vs. 12.0% vs. 8.5%), Bacteroidota (8.1% vs. 12.0% vs. 13.9%), Proteobacteria (3.3% vs. 0.9% vs. 6.1%) and Verrucomicrobiota (2.4% vs. 0.8% vs. 3.8%) (Supplementary Figure 5A). The dominant families in all groups were *Lachnospiraceae* (23.9% vs. 21.9% vs. 24.8%), *Ruminococcaceae* (13.2% vs. 16.9% vs. 11.2%), *Coriobacteriaceae* (11.2% vs. 8.9% vs. 5.3%), and *Bacteroidaceae* (4.8% vs. 5.0% vs. 6.1%) (Supplementary Figure 5B). At the phylum level, CRC patients were characterized by an increased relative abundance of Proteobacteria and Verrucomicrobiota, while Actinobacteriota was decreased compared to FAP patients (*P* ≤ .019) (Supplementary Figure 5C). Regarding the discriminating families, CRC patients had or tended to have the highest proportions of *Enterobacteriaceae*, *Veillonellaceae* and *Akkermansiaceae* (*P* ≤ .059) (Supplementary Figure 5D). FAP patients had a higher percentage of *Rikenellaceae* compared to FIT+ subjects and *Clostridia_UCG-014* compared to CRC patients (*P* ≤ .018). On the other hand, FIT+ subjects were mainly discriminated by higher proportions of *Erysipelotrichaceae* and *Butyricicoccaceae* (*P* ≤ .04).

The mucosa-associated microbiota of all groups was dominated by the phyla Firmicutes (mean relative abundance in the whole cohort, 39.8%), Proteobacteria (24.7%), Bacteroidota (23.0%) and Actinobacteriota (5.2%) (Supplementary Figure 6A). At the family level, *Lachnospiraceae* (20.2%), *Bacteroidaceae* (14.5%), *Pseudomonodaceae* (7.2%), and *Oxalobacteraceae* (6.9%) dominated the mucosa-associated microbiota of all groups (Supplementary Figure 6B). However, several differences emerged among the groups (Supplementary Figures 6C-E). CRC patients were enriched in Proteobacteria, as well as Fusobacteria and Planctomycetota, especially in the cancerous mucosa, compared to the other groups (*P* < .05). FAP P had a higher relative abundance of Firmicutes compared to the other groups (*P* < .05), and of Bacteroidota and Desulfobacterota compared to CRC tissues (*P* < .05). The FAP groups (*i.e.*, FAP P and FAP NM) were also characterized by lower proportions of Actinobacteriota, Patescibacteria, and Acidobacteriota compared to the other groups (*P* < .05). At the family level, we found an enrichment of *Corynebacteriaceae*, *Oxalobacteraceae*, and *Bacillaceae* in both CRC NM and CRC, compared to the other groups, and *Fusobacteriaceae*, *Micrococcaceae*, *Enterobacteriaceae*, and *Neisseriaceae* especially in CRC (*P* < .05). Conversely, *Ruminococcaceae* and *Oscillospiraceae* were less abundant in CRC (*P* < .05). The FAP P group was mainly distinguished by an overabundance of *Lachnospiraceae*, *Ruminococcaceae*, *Rikenellaceae*, *Acidaminococcaceae*, *Erysipelatoclostridiaceae*, and *Clostridia_UCG-01*4 (*P* < .05).

**Assessment of age as a potential confounding factor**

As the subject groups differed in mean age (mean (y/o) ± SEM; FIT+ = 61.6 ± 1.3, FAP = 32.7 ± 3.2, CRC = 67.3 ± 2.3;), with the FAP population being the youngest, we addressed the potential confounding effect of age in our cohort. To do this, we stratified FIT+ and CRC subjects into adults and elderly using 65 years as the threshold (9,10).

For the FAP group, which included no subjects over 65 years of age, we used the median age to have two numerically comparable groups. We then tested the differences in beta diversity (expressed as Bray-Curtis dissimilarity) between the two age groups thus defined for each subject group and microbial ecosystem (oral, faecal, and mucosal).

No significant differences were observed in oral samples (Supplementary Figure 7). For faecal samples, we only found a significant segregation between FAP subjects stratified by median age (28 years) (*P* = .048), with those under 28 years enriched in the *Subdoligranulum* genus (*P* = .002) (Supplementary Figure 8). It should be noted that *Subdoligranulum* was not previously identified as a discriminating taxon between subject groups, suggesting a greater impact of underlying pathology than age. For the mucosa-associated microbiota, we first evaluated age-related differences across all mucosal samples and then using NM for FIT+ and cancer or polyp samples for the CRC and FAP groups (Supplementary Figure 9). Notably, significant differences in beta diversity were only observed in FIT+ NM samples (*P* = .0065), with subjects under 65 years enriched in *Faecalibacterium* (*P* = .02). This taxon was also overrepresented in the FAP group (the youngest in our study) compared to FIT+ and CRC subjects, which limits its consideration as a possible marker of FAP disease.

**Harnessing Microbiome-Immune Crosstalk for Outcome Prediction**

To assess the association of microbiota with disease severity, we performed two different correlation analysis. One was based on the tumour-node-metastasis (TNM) system and the other on the Immunoscore system (IS). The latter seems to be more accurate to anticipate the outcome events in CRC than the TNM classification in colorectal cancers (3).

*TNM stage-based analysis.* We stratified CRC patients into two groups: Low T “0” and High T “1” based on the pathological pT (primary tumour) category/stage (0 = pT1 and pT2 (n=8), 1 = pT3 (n=7)), and compared the compositional structure of their mucosal microbiota. No significant differences emerged in beta diversity (expressed as Bray-Curtis dissimilarity) (Supplementary Figure 10A). However, the relative abundance of *Dialister* tended to be higher in the High T group, while that of *Prevotella*, *Streptococcus* and *Bifidobacterium* tended to be lower (*P* < .1) (Supplementary Figure 10B). We then sought for correlations between the relative abundances of the above taxa and WNT/b-catenin and PI3K/mTOR molecular markers. A positive correlation was found between *Prevotella* and cytosolic β-catenin in the Low T group (*P* =.02) (Supplementary Figure 10C).

*Immunoscore-based analysis.* To assess this, we calculated the IS in our CRC cohort as described in the method section above. Due to the low number of cases of our CRC cohort, for the microbiota analysis we decided to use the median density values as cut-off in order to have two balanced groups (n=6 for I0-I1-I2, n=4 for I3-I4). Principal Coordinates Analysis of Bray-Curtis dissimilarity showed no significant separation (Adonis P > .1) (Supplementary Figure 11A). Regarding taxonomy, *Bacteroides* and *Butyricicoccus* tended to be higher in the high IS group compared to the low IS group (Wilcoxon test P = .07 and P = .09, respectively) (Supplementary Figure 11B). When searching for correlations between these genera and WNT/b-catenin and PI3K/mTOR molecular markers, we found that *Bacteroides* correlated positively with the expression of *AXIN2* (rho: 0.97; *P* = .001) and c*MYC* (rho: 0.97; *P* = .001) in the low IS group (Supplementary Figure 11C).

*Butyricicoccus* is known to produce butyrate, a short-chain fatty acid that may play a critical role in the local activation of the immune system, promoting cell proliferation and facilitating tumour infiltration. In particular, butyrate can stimulate the recruitment of immune cells (such as T cells and NK cells) to the tumour site, enhance the activation of cytotoxic T cells and increase their antitumour function (11,12). In addition, butyrate has been shown to suppress the production of pro-inflammatory cytokines (such as TNF-alpha and IL-6) by tumour-associated macrophages (13–15). This effect may lead to a reduction in the recruitment of immunosuppressive myeloid-derived suppressor cells, thereby creating a more favourable immune environment for antitumour activity (16,17). No less importantly, butyrate has also shown epigenetic activity by inhibiting histone deacetylase. This inhibition affects the gene expression of immune cells and colonocytes, thereby influencing immune system activity and cancer progression (18,19). On the other hand, lipopolysaccharide expressed on the surface of *Bacteroides* or released from outer membrane vesicles is known to activate immune cells via Toll-like receptors, affecting their function and influencing lymphocyte recruitment, tumour infiltration and immune surveillance activity (20,21). Furthermore, several further pathogenic mechanisms, have been associated to members of *Bacteroides* to favour an immunosuppressed microenvironment that helps the tumour progression (22). Among those, the ability to induce mucus layer degradation (23) or modulate IL-10 by the *B. fragilis* polysaccharide A (PSA) (22). Moreover, a direct relationship between TLR activation, tumour-derived WNT ligands and TLR-activated monocytes secreting IL-10 has been previously described in lung cancers (24), together with the *B. fragilis* zinc-dependent metalloprotease toxin found to interact with E-cadherin, leading the disruption of intercellular junctions, and promoting the activation of Wnt signaling-associated proto-oncogenes, such as cMYC (25,26).

These evidences may support the relevance of *Bacteroides* in correlation with the Wnt signalling and the immunoscore as potential predictive biomarkers for CRC prognosis, since low immunoscore is associated with poor overall survival and disease free survival in patients with CRC (27,28).

**BIBLIOGRAPHY**

1. Bankhead P, Loughrey MB, Fernández JA, Dombrowski Y, McArt DG, Dunne PD, et al. QuPath: Open source software for digital pathology image analysis. Sci Rep. 2017;7(1):16878.

2. Anitei M-G, Zeitoun G, Mlecnik B, Marliot F, Haicheur N, Todosi A-M, et al. Prognostic and predictive values of the immunoscore in patients with rectal cancer. Clin cancer Res an Off J Am Assoc Cancer Res. 2014 Apr;20(7):1891–9.

3. Mlecnik B, Tosolini M, Kirilovsky A, Berger A, Bindea G, Meatchi T, et al. Histopathologic-based prognostic factors of colorectal cancers are associated with the state of the local immune reaction. J Clin Oncol Off J Am Soc Clin Oncol. 2011 Feb;29(6):610–8.

4. Galon J, Pagès F, Marincola FM, Angell HK, Thurin M, Lugli A, et al. Cancer classification using the Immunoscore: a worldwide task force. J Transl Med. 2012 Oct;10:205.

5. Galon J, Pagès F, Marincola FM, Thurin M, Trinchieri G, Fox BA, et al. The immune score as a new possible approach for the classification of cancer. Vol. 10, Journal of translational medicine. England; 2012. p. 1.

6. D’Amico F, Biagi E, Rampelli S, Fiori J, Zama D, Soverini M, et al. Enteral Nutrition in Pediatric Patients Undergoing Hematopoietic SCT Promotes the Recovery of Gut Microbiome Homeostasis. Vol. 11, Nutrients. 2019.

7. McIver LJ, Abu-Ali G, Franzosa EA, Schwager R, Morgan XC, Waldron L, et al. bioBakery: a meta’omic analysis environment. Bioinformatics. 2018 Apr;34(7):1235–7.

8. Rognes T, Flouri T, Nichols B, Quince C, Mahé F. VSEARCH: a versatile open source tool for metagenomics. PeerJ. 2016;4:e2584.

9. Ghosh TS, Shanahan F, O’Toole PW. The gut microbiome as a modulator of healthy ageing. Nat Rev Gastroenterol Hepatol. 2022;19(9):565–84.

10. Biagi E, Nylund L, Candela M, Ostan R, Bucci L, Pini E, et al. Through Ageing, and Beyond: Gut Microbiota and Inflammatory Status in Seniors and Centenarians. PLoS One. 2010 May 17;5(5):e10667.

11. Danne C, Sokol H. Butyrate, a new microbiota-dependent player in CD8+ T&#xa0;cells immunity and cancer therapy? Cell Reports Med. 2021 Jul 20;2(7).

12. Luu M, Riester Z, Baldrich A, Reichardt N, Yuille S, Busetti A, et al. Microbial short-chain fatty acids modulate CD8+ T cell responses and improve adoptive immunotherapy for cancer. Nat Commun. 2021;12(1):4077.

13. Liu H, Wang J, He T, Becker S, Zhang G, Li D, et al. Butyrate: A Double-Edged Sword for Health? Adv Nutr. 2018;9(1):21–9.

14. Mann ER, Lam YK, Uhlig HH. Short-chain fatty acids: linking diet, the microbiome and immunity. Nat Rev Immunol. 2024.

15. Zhai S, Qin S, Li L, Zhu L, Zou Z, Wang L. Dietary butyrate suppresses inflammation through modulating gut microbiota in high-fat diet-fed mice. FEMS Microbiol Lett. 2019 Jul 1;366(13):fnz153.

16. Xiao T, Zhang P, Feng T, Lu K, Wang X, Zhou S, et al. Butyrate functions in concert with myeloid-derived suppressor cells recruited by CCR9 to alleviate DSS-induced murine colitis. Int Immunopharmacol. 2021;99:108034.

17. Xiao T, Sun M, Chang Y, Kang J, Zhao C, Zhu R, et al. Butyrate impeded the conscription of MDSCs to reduce CAC formation by blocking the TLR2 signaling pathway. J Funct Foods. 2022;99:105344.

18. Mohamed Elfadil O, Mundi MS, Abdelmagid MG, Patel A, Patel N, Martindale R. Butyrate: More Than a Short Chain Fatty Acid. Curr Nutr Rep. 2023;12(2):255–62.

19. Chriett S, Dąbek A, Wojtala M, Vidal H, Balcerczyk A, Pirola L. Prominent action of butyrate over β-hydroxybutyrate as histone deacetylase inhibitor, transcriptional modulator and anti-inflammatory molecule. Sci Rep. 2019;9(1):742.

20. Sulit AK, Daigneault M, Allen-Vercoe E, Silander OK, Hock B, McKenzie J, et al. Bacterial lipopolysaccharide modulates immune response in the colorectal tumor microenvironment. npj Biofilms Microbiomes. 2023;9(1):59.

21. Song W, Tiruthani K, Wang Y, Shen L, Hu M, Dorosheva O, et al. Trapping of Lipopolysaccharide to Promote Immunotherapy against Colorectal Cancer and Attenuate Liver Metastasis. Adv Mater. 2018 Dec 1;30(52):1805007.

22. Zafar H, Saier MHJ. Gut Bacteroides species in health and disease. Gut Microbes. 2021;13(1):1–20.

23. Desai MS, Seekatz AM, Koropatkin NM, Kamada N, Hickey CA, Wolter M, et al. A Dietary Fiber-Deprived Gut Microbiota Degrades the Colonic Mucus Barrier and Enhances Pathogen Susceptibility. Cell. 2016 Nov;167(5):1339-1353.e21.

24. Martín-Medina A, Cerón-Pisa N, Martinez-Font E, Shafiek H, Obrador-Hevia A, Sauleda J, et al. TLR/WNT: A Novel Relationship in Immunomodulation of Lung Cancer. Vol. 23, International Journal of Molecular Sciences. 2022.

25. Wu S, Morin PJ, Maouyo D, Sears CL. Bacteroides fragilis enterotoxin induces c-Myc expression and cellular proliferation. Gastroenterology. 2003 Feb;124(2):392–400.

26. Wu S, Rhee K-J, Zhang M, Franco A, Sears CL. Bacteroides fragilis toxin stimulates intestinal epithelial cell shedding and gamma-secretase-dependent E-cadherin cleavage. J Cell Sci. 2007 Jun;120(Pt 11):1944–52.

27. Sun G, Dong X, Tang X, Qu H, Zhang H, Zhao E. The prognostic value of immunoscore in patients with colorectal cancer: A systematic review and meta-analysis. Cancer Med. 2019 Jan 1;8(1):182–9.

28. Mlecnik B, Torigoe T, Bindea G, Popivanova B, Xu M, Fujita T, et al. Clinical Performance of the Consensus Immunoscore in Colon Cancer in the Asian Population from the Multicenter International SITC Study. Cancers (Basel). 2022 Sep;14(18).

**TABLES**

**Supplementary Table 1.** Sequences with relative length and annealing temperature of APC exons primers. A.T.: annealing temperature. Reference genome GRCh37/hg19.

| Patient ID | Amplicon | Forward Primer (5’ 🡪 3’) | Reverse Primer (5’🡪 3’) | Size (bp) | A.T. |
| --- | --- | --- | --- | --- | --- |
| PT 04, 09 | Exon 3 | CTCAGCATACTTAAATGTCAAGA | AACTGATACCAACACCCAAAT | 280 | 58 °C |
| PT 18, 20 | Exon 4 | TCAGTCATGTATATTTGTGGTT | GTAAGTGCAATCAATAACATGC | 487 | 60 °C |
| PT 07, 13, 14, 15 | Exon 5 | TCTTTTAAGGATGATTACCAG | TATTCAGGCCTAAAGTTGGG | 494 | 58 °C |
| PT 03 | Exon 9 | CAGACACTTCATTTGGAGTACC | AGTAGAGATGGGGTTTTGCC | 369 | 60 °C |
| PT 17 | Exon 10 | TGGTGATGATACATAGATTTTGA | GAGTGAATGATGTTGTGGAGT | 444 | 60 °C |
| PT 16, 19 | Exon 12 | TGTAGCTTATAATTCTAAAGGCA | AAAGATAAGCGAATGTGAAGC | 433 | 58 °C |
| PT 05, 06 | Exon 14 | CACGGCTAGCCAGAATTTCT | ACACAGGTAAGAAATTAGGA | 335 | 58 °C |
| PT 08  PT 01, 02 | Exon 16 | TAGAAGTTTGGAGAGAGAACG  TGGAGAACTAGATACACCAATA | TGACCTCTTTTACCATAACCA  CGTTCACTATAATTGGTAGGC | 387  384 | 60 °C  56 °C |

**Supplementary Table 2.** Germline and somatic APC mutations in FAP patients. NM: normal appearing mucosa, P: polyp.

Variants were classified as pathogenic or likely pathogenic on ClinVar database, except for *APC* (c.292_300delAGCCGGGAA) and *NOTCH* (c.4742C>T) which were described as variants of uncertain significance (VUS). ͌ Variants identified with the custom Ion AmpliSeq On-Demand Panel; ^º^ Variants identified with the Ion Ampliseq Cancer HotSpot Panel; * Variant not confirmed by Sanger sequencing analysis.

|  | | | **Germline *APC* mutation** | | | **Somatic mutations** | | | |
| --- | --- | --- | --- | --- | --- | --- | --- | --- | --- |
| *ID* | *Gender* | *Age* | *Coding region* | *Protein* | *Function* | *Gene* | *Function* | *Coding* | *Protein* |
| 01 NM | Male | 19 | c.3202-3205delTCAA | p.Ser1068fs | frameshift | *APC* ^º^ | nonsense | c.3907C>T | p.Gln1303Ter |
| 01 P |  |  | c.3202-3205delTCAA | p.Ser1068fs | frameshift | *APC*  ͌ | nonsense | c.4216C>T | p.Gln1406Ter |
| 02 NM | Female | 23 | c.3202-3205delTCAA | p.Ser1068fs | frameshift |  |  |  |  |
| 02 P |  |  | c.3202-3205delTCAA | p.Ser1068fs | frameshift |  |  |  |  |
| 03 NM | Male | 19 | c.847C>T | p.Arg283Ter | nonsense |  |  |  |  |
| 03 P |  |  | c.847C>T | p.Arg283Ter | nonsense |  |  |  |  |
| 04 NM | Female | 57 | c.147_150delACAA | p.Lys49AsnfsX19 | frameshift | *APC* ^º^ | missense | c.2627G>A | p.Arg876Gln |
| 05 NM | Female | 23 | c.1690C>T | p.Arg564Ter | nonsense |  |  |  |  |
| 05 P |  |  | c.1690C>T | p.Arg564Ter | nonsense | *APC*  ͌ ^º^ | frameshiftDeletion | c.3927_3931delAAAGA | p.Glu1309AspfsTer4 |
| 06 NM | Male | 31 | c.1690C>T | p.Arg564Ter | nonsense |  |  |  |  |
| 06 P |  |  | c.1690C>T * | p.Arg564Ter * | Nonsense * | *APC* ^º^ | frameshiftDeletion | c.3927_3931delAAAGA | p.Glu1309AspfsTer4 |
| 07 NM | Male | 44 | c.423-2A>T | p.? | Splicing variant |  |  |  |  |
| 07 P |  |  | c.423-2A>T | p.? | Splicing variant | *APC* ͌ | nonsense | c.3845C>A | p.Ser1282Ter |
| 08 NM | Male | 31 | c.2833delA | p.Arg945GlyfsTer10 | frameshift |  |  |  |  |
| 08 P |  |  | c.2833delA | p.Arg945GlyfsTer10 | frameshift |  |  |  |  |
| 09 NM | Female | 26 | c.147_150delACAA | p.Lys49AsnfsTer19 | frameshift |  |  |  |  |
| 13 NM | Male | 54 | c.531+1G>A | p.? | Splice donor variant |  |  |  |  |
| 13 P |  |  | c.531+1G>A | p.? | Splice donor variant | *APC*  ͌ ^º^ *KRAS* ^º^ | nonsense missense | c.4396G>T c.38G>A | p.Gly1466Ter  p.Gly13Asp |
| 14 NM | Male | 54 | c.531+1G>A | p.? | Splice donor variant |  |  |  |  |
| 14 P |  |  | c.531+1G>A | p.? | Splice donor variant | *APC* ͌ | nonsense | c.4132C>T | p.Gln1378Ter |
| 15 NM | Male | 44 | c.423-1G>A | p.? | Splice acceptor variant | *APC* ^º^ | nonsense | c.4132C>T | p.Gln1378Ter |
| 15 P |  |  | c.423-1G>A | p.? | Splice acceptor variant | *APC* ͌ | frameshift | c.3866_3870delGTAAT | p.Cys1289SerfsTer10 |
| 16 NM | Male | 20 | c.1495C>T | p.Arg499Ter | nonsense |  |  |  |  |
| 16 P |  |  | c.1495C>T | p.Arg499Ter | nonsense | *APC*  ͌ ^º^ | frameshiftDeletion | c.4350delA | p.Glu1451LysfsTer22 |
| 17 NM | Female | 30 | c.940delA | p.Met314TrpfsTer22 | frameshift |  |  |  |  |
| 17 P |  |  | c.940delA | p.Met314TrpfsTer22 | frameshift | *APC* ^º^  *APC* ͌ | nonsense  nonsense | c.4348C>T  c.646C>T | p.Arg1450Ter  p. Arg216Ter |
| 18 NM | Male | 24 | c.287_299delinsGG | p.Tyr96TrpfsTer39 | frameshift |  |  |  |  |
| 18 P |  |  | c.287_299delinsGG | p.Tyr96TrpfsTer39 | frameshift | *APC* ͌ | in frame | c.292_300delAGCCGGGAA | p.Ser98_Glu100del |
| 19 NM | Male | 23 | c.1495C>T | p.Arg499Ter | nonsense |  |  |  |  |
| 19 P |  |  | c.1495C>T | p.Arg499Ter | nonsense | *APC* ͌ | frameshift | c.4245_4246insT | p.Gly1416TrpfsTer7 |
| 20 NM | Female | 33 | c.287_299delinsGG | p.Tyr96TrpfsTer39 | frameshift | *NOTCH1* ^º^ | missense | c.4742C>T | p.Pro1581Leu |
| 20 P |  |  | c.287_299delinsGG | p.Tyr96TrpfsTer39 | frameshift | *APC* ͌ | in frame | c.292_300delAGCCGGGAA | p.Ser98_Glu100del |

**Supplementary Table 3.** Clinicopathological features of patient with sporadic CRC.

Variants were classified as pathogenic or likely pathogenic on ClinVar database, except for *PIK3CA* (c.3197C>T), *TP53* (c.472_477dup) and *ERBB4* (c.1073A>T) which were described as VUS. The function of *APC ^o^* variants is not yet available on ClinVar database, as they are common types of mutations in germline *APC* mutated settings, they can be considered likely pathogenic. MSI: microsatellite instable; MSS: microsatellite stable; NA: not available.

| **CRC Patients** | | | | | **Somatic mutations** | | | |
| --- | --- | --- | --- | --- | --- | --- | --- | --- |
| *ID Patients* | *Gender* | *Age* | *Tumor stage* | *MMR Protein Status* | *Gene* | *Function* | *Coding* | *Protein* |
| 02 CRC | Female | 68 | pT3 N1A MX | MSS | *APC ^o^*  *APC ^o^*  *TP53*  *TP53* | frameshift  frameshift  splicing  nonsense | c.2736_2737insA  c.4225_4226insGAAC  c.919+1G>A  c.991C>T | p.His913ThrfsTer6  p.Pro1409ArgfsTer15  p.?  p.Gln331Ter |
| 03 CRC | Female | 64 | pT3 N0 | MSS | *PIK3CA*  *APC KRAS GNAS* | missense  frameshift  missense  missense | c.1633G>A  c.2926_2927insA c.35G>A c.601C>T | p.Glu545Lys  p.Arg976LysfsTer9 p.Gly12Asp p.Arg201Cys |
| 04 CRC | Female | 77 | pT3 N0 MX | NA | *APC ^o^*  *APC ^o^*  *ATM*  *TP53* | nonsense  frameshift  nonsense  inframe | c.784G>T  c.4245delT  c.6415G>T  c.472_477dup | p.Glu262Ter  p.Ser1415ArgfsTer4  p.Glu2139Ter  p.Arg158_Ala159dup |
| 05 CRC | Male | 63 | pT2 N0 MX | MSS | *APC PTEN KRAS SMAD4* | frameshift  nonsense  missense  missense | c.4463delT c.697C>T c.35G>A c.1082G>A | p.Leu1488TyrfsTer19 p.Arg233Ter p.Gly12Asp p.Arg361His |
| 06 CRC | Male | 53 | pT2 N0 | MSS |  |  |  |  |
| 07 CRC | Female | 67 | pT2 N0 MX | MSI; loss of MSH2 e MSH6 |  |  |  |  |
| 08 CRC | Male | 63 | pT3 N0 MX | MSS |  |  |  |  |
| 09 CRC | Male | 80 | pT1 N0 MX | MSI; loss of MLH1 e PMS2 | *BRAF*  *MSH3 PIK3CA* | missense  frameshift  missense | c.1799T>A  c.1148delA c.3197C>T | p.Val600Glu  p.Lys383ArgfsTer32  p.Ala1066Val |
| 10 CRC | Male | 67 | pT3 N1 R0 | MSS | *APC KRAS TP53* | nonsense missense  missense | c.3907C>T c.35G>A c.524G>A | p.Gln1303Ter p.Gly12Asp p.Arg175His |
| 11 CRC | Female | 72 | pT2 N0 MX | MSI; loss of MLH1 e PMS2 | *ERBB4 BRAF*  *MSH3* | missense  missense  frameshift | c.1073A>T c.1799T>A  c.1148delA | p.Asn358Ile p.Val600Glu  p.Lys383ArgfsTer32 |
| 12 CRC | Male | 58 | pT3 N0 | MSS | *IDH1 BRAF TP53* | missense missense  missense | c.394C>T c.1799T>A c.724T>G | p.Arg132Cys p.Val600Glu p.Cys242Gly |
| 13 CRC | Male | 77 | pT2 N0 MX | MSS | *APC ^o^*  *TP53 TP53* | nonsense  missense  missense | c.4182_4183insT  c.524G>A c.404G>T | p.Ser1395Ter  p.Arg175His p.Cys135Phe |
| 14 CRC | Female | 51 | pT2 | MSS | *APC TP53* | frameshift  unknown | c.3911delT c.994-1G>A | p.Ile1304LysfsTer4 p.? |
| 15 CRC | Male | 79 | pT1 N0 MX | MSS | *PIK3CA APC KRAS GNAS* | missense  frameshift  missense  missense | c.1633G>A c.4474delG c.35G>C c.601C>T | p.Glu545Lys p.Ala1492ProfsTer15 p.Gly12Ala  p.Arg201Cys |
| 16 CRC | Male | 70 | pT3, N0 MX | MSS | *PIK3CA*  *APC TP53* | missense  nonsense  missense | c.1258T>C  c.847C>T c.524G>A | p.Cys420Arg  p.Arg283Ter p.Arg175His |

**Supplementary Table 4.** General features of FIT+, FAP and CRC patients.

| ***FIT+ - ID Patients*** | ***Age*** | ***Dietary status*** | ***Physical Activity*** | ***Weight (Kg)*** | ***Height (cm)*** | ***Comorbidities*** | ***Ongoing therapies*** |
| --- | --- | --- | --- | --- | --- | --- | --- |
| 02 FIT+ | 57 | Mixed | Moderate | 76 | 175 | Hypertension | Lexotan |
| 03 FIT+ | 63 | Mixed | Moderate | 98 | 180 | Dyslipidemia | Cardioaspirin, dilatrend, goltor, eskim |
| 04 FIT+ | 59 | Mixed | Moderate | 83 | 173 | None | None |
| 05 FIT+ | 50 | Mixed | Moderate | 78 | 177 | None | None |
| 06 FIT+ | 64 | Mixed | Sedentary | 113 | 160 | Spondyloarthritis, hypertension, atrial fibrillation | Xalelto, Biologico per spondiloartrite; hypertension therapy |
| 07 FIT+ | 65 | Mixed | Moderate | 63 | 158 | Periarthritis, arterial hypertension | NA |
| 08 FIT+ | 68 | Mixed | Moderate | 64 | 163 | Hypothyroidism, mitral prolapse, osteoporosis | NA |
| 09 FIT+ | 63 | Mixed | Moderate | 60 | 167 | None | None |
| 10 FIT+ | 68 | Mixed | Moderate | 58 | 158 | Hypercholesterolemia, diabetes | Metformin, Statin, Omega-3, Vitamin D |
| 11 FIT+ | 63 | Mixed | Sedentary | 62 | 173 | None | Nessuna |
| 12 FIT+ | 65 | Mixed | Sedentary | 60 | 156 | Hypertension, depression/anxiety | Cotareg, Lorazepam, Daparox |
| 13 FIT+ | 54 | Mixed | Sedentary | 87 | 179 | None | Eutirox |
| 14 FIT+ | 61 | Mixed | Moderate | 73 | 167 | None | None |
| 15 FIT+ | 54 | Mixed | Sedentary | 69 | 155 | None | None |
| 16 FIT+ | 59 | Mixed | Moderate | 55 | 168 | Hypercholesterolemia | Statin |
| 17 FIT+ | 65 | Mixed | Moderate | 78 | 173 | Dyslipidemia | Omega3, normolipid 5 |
| 18 FIT+ | 69 | Mixed | Moderate | 67 | 155 | Hypercholesterolemia, hypertension | Statina, coverlam |
| ***FAP - ID Patients*** |  | ***Dietary status*** | ***Physical Activity*** | ***Weight (Kg)*** | ***Height (cm)*** | ***Comorbidities*** | ***Ongoing therapies*** |
| 01 FAP | 19 | Mixed | Moderate | 101 | 197 | None | None |
| 02 FAP | 23 | Mixed | Moderate | 87 | 177 | None | None |
| 03 FAP | 19 | Mixed | Sedentary | 47 | NA | None | None |
| 04 FAP | 57 | Mixed | Moderate | 80 | NA | Hypertension/GERD | Hypertension therapy |
| 05 FAP | 23 | Mixed | Moderate | 55 | 165 | None | None |
| 06 FAP | 31 | Mixed | Moderate | 82 | 170 | None | None |
| 07 FAP | 44 | Mixed | Sedentary | 113 | 186 | None | None |
| 08 FAP | 31 | Mixed | Moderate | 60 | 160 | None | None |
| 09 FAP | 26 | Mixed | Moderate | 70 | 165 | Hashimoto's thyroiditis | Eutirox, contraceptive pill |
| 13 FAP | 54 | Vegetarian | Sedentary | 80 | 186 | NA | NA |
| 14 FAP | 54 | Mixed | Moderate | 87 | 187 | NA | NA |
| 15 FAP | 44 | Mixed | Moderate | 105 | 190 | NA | NA |
| 16 FAP | 20 | Mixed | Moderate | 85 | 187 | NA | NA |
| 17 FAP | 30 | Vegetarian | Moderate | 57 | 160 | NA | NA |
| 18 FAP | 24 | Mixed | Moderate | 100 | 175 | NA | NA |
| 19 FAP | 23 | Mixed | Moderate | 75 | 185 | NA | NA |
| 20 FAP | 33 | Mixed | Moderate | 55 | 160 | NA | NA |
| ***CRC - ID Patients*** |  | ***Dietary status*** | ***Physical Activity*** | ***Weight (Kg)*** | ***Height (cm)*** | ***Comorbidities*** | ***Ongoing therapies*** |
| 02 CRC | 68 | Mixed | Moderate | 90 | 167 | None | Eutirox; Vitamin D; Movicol |
| 03 CRC | 64 | Mixed | Moderate | 66 | 172 | Psoriasis | None |
| 04 CRC | 77 | Mixed | Moderate | 59 | 158 | Dyslipidemia, hypertension, osteopenia, anxious depression | Losartan and diuretics, atorvastatin, levosurpride |
| 05 CRC | 63 | Mixed | Sedentary | 88 | 178 | Diabetes | Insulin |
| 06 CRC | 53 | Mixed | Moderate | 85 | 179 | Seborrheic dermatitis | None |
| 07 CRC | 67 | Mixed | Moderate | 62 | 164 | Factor V Leiden mutation | NA |
| 08 CRC | 63 | Mixed | Moderate | 104 | 177 | Hypertension | Losartan |
| 09 CRC | 80 | Mixed | Sedentary | 72 | 162 | None | None |
| 10 CRC | 67 | Mixed | Moderate | 75 | 172 | Diabetes, hypertension, arrhythmia, | Eutirox, Diamicron, Vaporetic, Rytmonorm, Syntaris, Solosa, Totalip |
| 11 CRC | 72 | Mixed | Moderate | 64 | 160 | Hyperuricemia, hypertension, depression/anxiety | Ramipril, Zoloft, Cardioaspirin, Vitamin D, Saflutan, Bisoprol, Allopurinol |
| 12 CRC | 58 | Mixed | Moderate | 72 | 177 | NA | NA |
| 13 CRC | 77 | Mixed | Intense | 65 | 169 | NA | NA |
| 14 CRC | 51 | Mixed | Intense | 56 | 165 | NA | NA |
| 15 CRC | 79 | Mixed | Moderate | 89 | 167 | Hypertension, hypertriglyceridemia | Losartan; Cardirene; Olevia |
| 16 CRC | 70 | Mixed | Sedentary | 81 | 179 | Diabetes | Metformin, ramipril, metoprolol, ozempic, cardioaspirin, insulin, ezetimibe |

**Supplementary Table 5.** Detailed description of the calculation required for the relative frequencies distribution method. The example of Ki67.

1. Raw data in % for each case/group.

1. Ki67-Score for each case/group.

1. Frequences classes with Absolute and Relative Frequency Distribution.

**Supplementary Table 6.** Description of the procedure used for the Immunoscore (IS) analysis. Each tumour region was categorized into High and Low density for each marker in each tumour region according to the median cut-off values. Patients were then stratified according to a score ranging from I0 to I4 (2–5). Finally, patients with I0-I1-I2 were considered with Low IS (group: Low) and those with I3-I4 were considered with High IS (group: High) for the subsequentially correlation analysis. Patients 05, 07, 09 and 14 were excluded because their tissues were not suitable for the IS analysis.

|  | **CD3+** | | | | **CD8+** | | | | **IS** | **Group** |
| --- | --- | --- | --- | --- | --- | --- | --- | --- | --- | --- |
|  | *CT* | | *IM* | | *CT* | | *IM* | |  |  |
|  | Positive cells/mm^2^ | Immune Infiltration | Positive cells/mm^2^ | Immune Infiltration | Positive cells/mm^2^ | Immune Infiltration | Positive cells/mm^2^ | Immune Infiltration |  |  |
| PT02 CRC | 621 | Low | 1945 | High | 56 | Low | 481 | Low | I1 | Low |
| PT03 CRC | 639 | Low | 4647 | High | 379 | High | 2950 | High | I3 | High |
| PT04 CRC | 792 | High | 4106 | High | 228 | Low | 1373 | High | I3 | High |
| PT06 CRC | 654 | High | 1007 | Low | 95 | Low | 268 | Low | I1 | Low |
| PT08 CRC | 639 | Low | 1062 | Low | 243 | High | 395 | Low | I1 | Low |
| PT10 CRC | 402 | Low | 1562 | High | 76 | Low | 512 | Low | I1 | Low |
| PT11 CRC | 444 | Low | 1124 | Low | 610 | High | 1255 | High | I2 | Low |
| PT12 CRC | 768 | High | 755 | Low | 172 | Low | 184 | Low | I1 | Low |
| PT13 CRC | 1370 | High | 2428 | High | 592 | High | 1148 | High | I4 | High |
| PT15 CRC | 671 | High | 1018 | Low | 456 | High | 569 | High | I3 | High |
| Median | 647 |  | 1343 |  | 235 |  | 541 |  |  |  |

**FIGURES**

**Supplementary Figure 1. Stemness and proliferation markers expression in FIT+, FAP and CRC including CRC NM tissues**. A) Expression levels of cytosolic and nuclear β-catenin, nuclear Ki67; B) cytosolic p-p70S6K and p-S6R. Values are shown as median with 95% CI, scale bars: 100 µm, magnification: 200x. Ten independent fields were quantified for CRC tissues while six to eight for FIT+ and FAP tissues; n = 14 (FIT+), 13 (FAP NM, FAP P, CRC NM), 15 (CRC). C-E) RNA expression levels of Wnt/β-catenin and mTOR downstream effectors. Values are shown as mean ± SD; n = 17 (FIT+), 16 (FAP NM), 13 (FAP P), 15 (CRC NM, CRC). * *P* < .05, ** *P* < .001, *** *P* < .001, **** *P* < .0001.


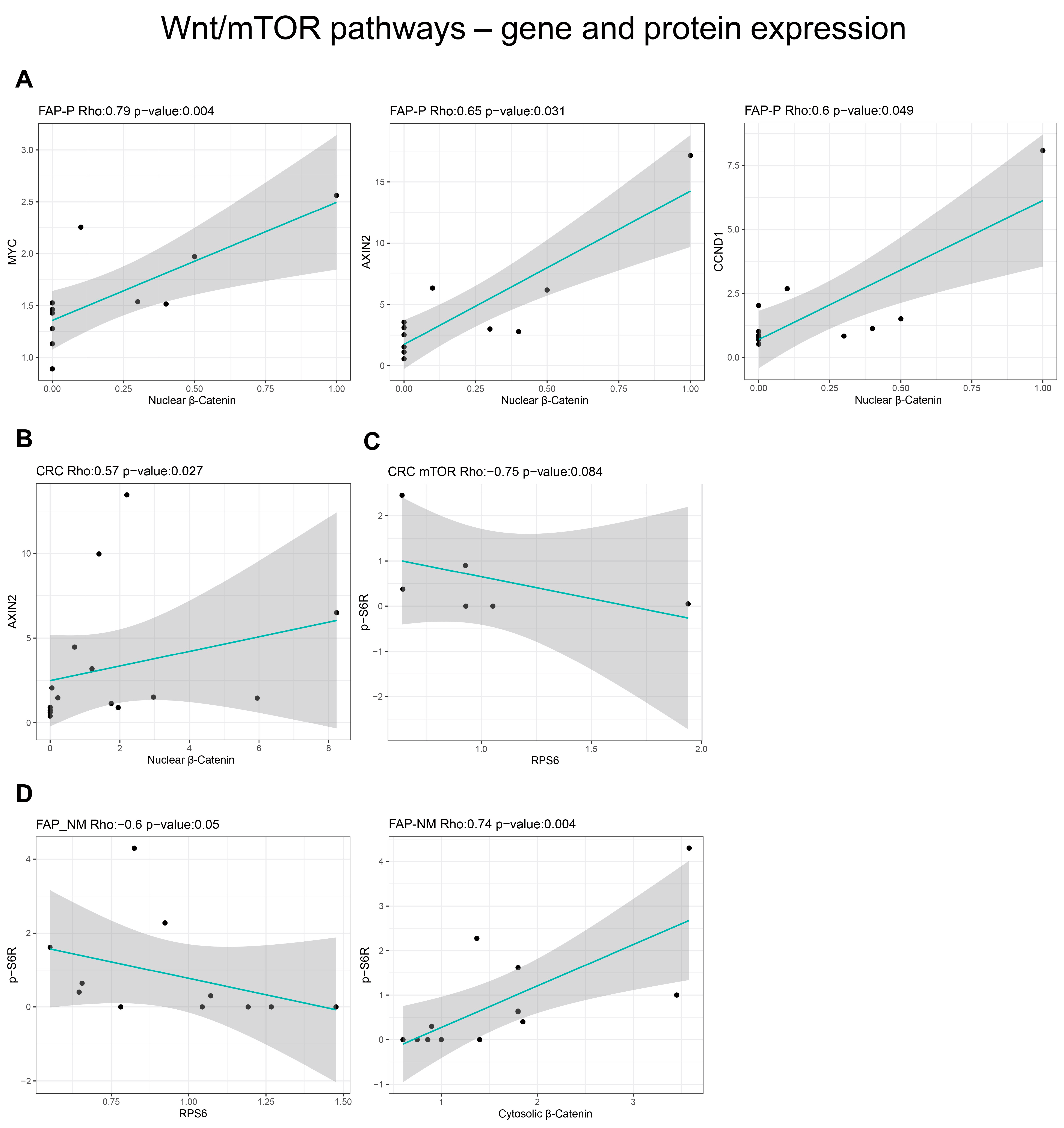
**Supplementary Figure 2. Scatterplots reporting significant correlations among downstream expression effectors of Wnt/β-catenin and PI3K/mTOR pathways**. Scatterplots reported results of the Spearman correlation test used to determine correlations among Wnt/mTOR proteins (*i.e.* Nuclear β-Catenin, Cytosolic β-Catenin and p-S6R) and genes (*i.e.* *MYC*, *AXIN2*, *CCND1* and *RPS6*) in FAP and CRC population. Significant correlations of Nuclear β-Catenin levels with the Wnt target genes (A) in FAP adenomatous polyps and (B) CRC tissues. (C) Significant correlations in CRC tissues sub-grouped by somatic mutation in PI3K/mTOR. (D) Significant correlation in FAP normal mucosal tissue. Only significant Spearman’s correlations (*P* < .05) with |rho| > 0.3 are shown.


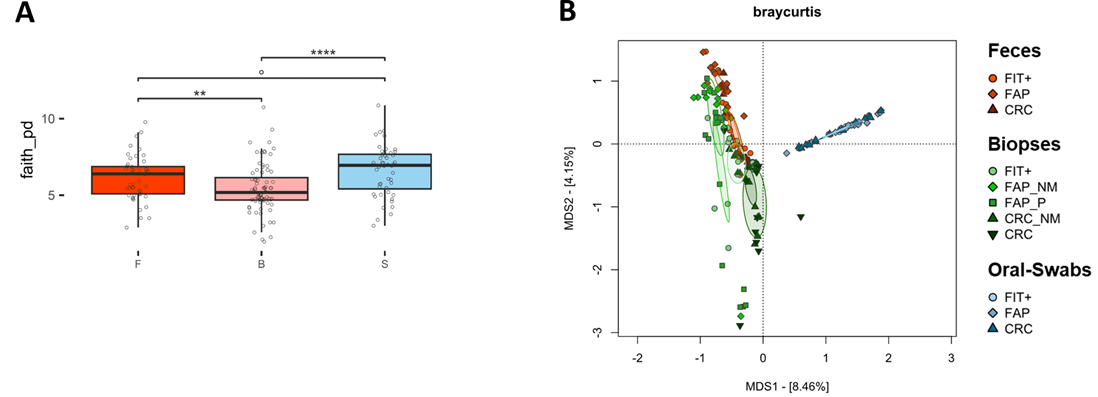


**Supplementary Figure 3. Alpha and beta diversity of the oral, faecal and mucosa-associated microbiota in the whole cohort.** A) Distribution of alpha diversity, calculated using Faith’s phylogenetic diversity, in the oral (S), faecal (F) and mucosa-associated microbial profiles (B) of the whole cohort. Wilcoxon test, ° *P* < .1, ** *P* < .01, **** *P* < .0001. B) PCA of Bray-Curtis dissimilarity between microbial profiles of oral swabs, faeces and biopsies from FAP, CRC and FIT+ subjects. Biopsies included adenomatous polyps from FAP patients (FAP P), cancerous mucosa from CRC patients (CRC) and normal mucosa from FAP (FAP NM), CRC (CRC NM) and FIT+ subjects (FIT+). Ellipses include a 95% confidence area based on the standard error of the weighted average of sample coordinates. A significant separation was found between all groups (Adonis, *P* = .0001).


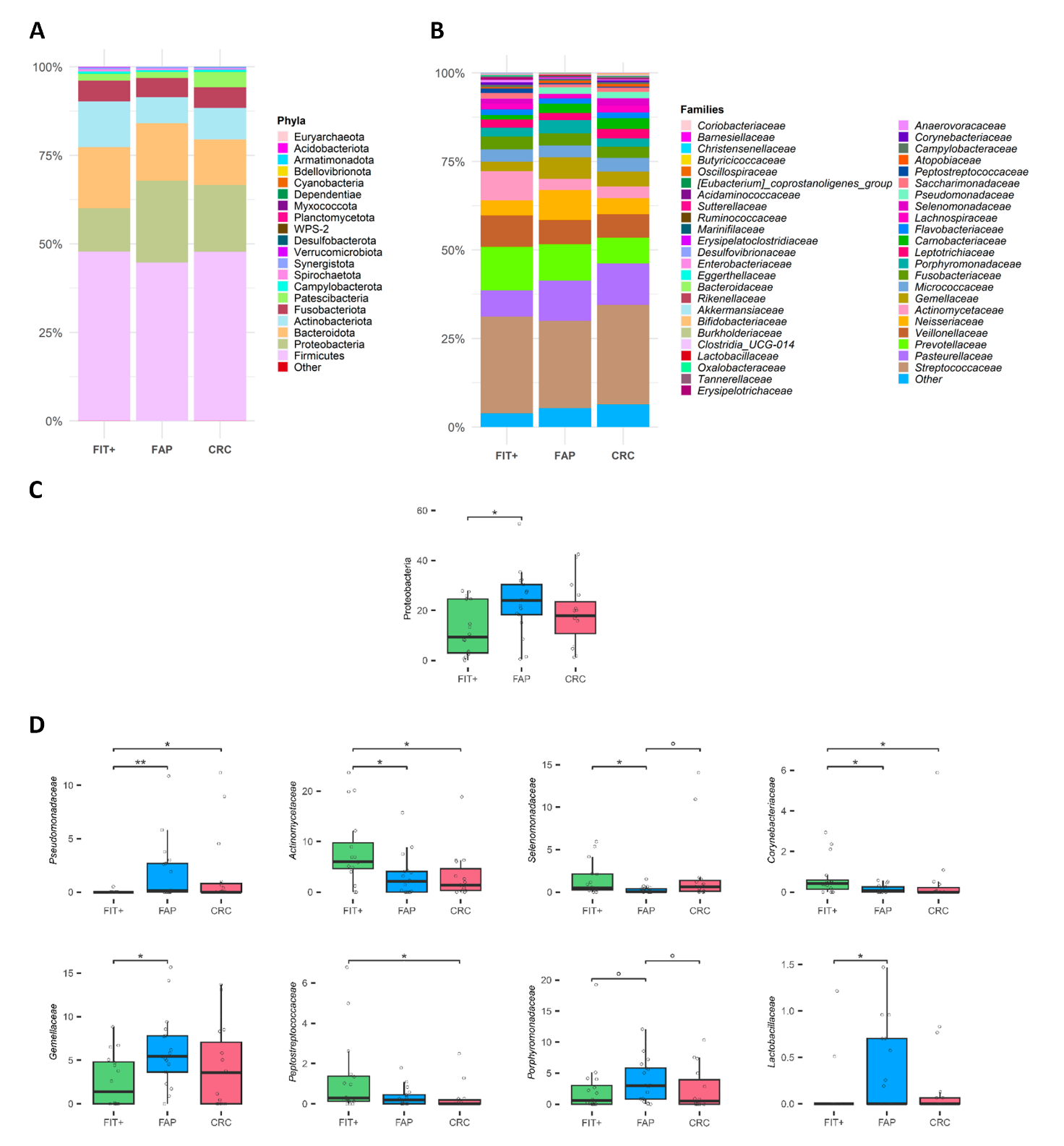


**Supplementary Figure 4. Phylum- and family-level compositional differences in the oral microbiota between FAP, CRC and FIT+ subjects.** Relative abundance distribution of bacterial phyla (A) and families (B) differentially represented between groups. Mean relative abundances of the major phyla (C) and families (D). Wilcoxon test, ° *P* < .1; * *P* < .05; ** *P* < .01.


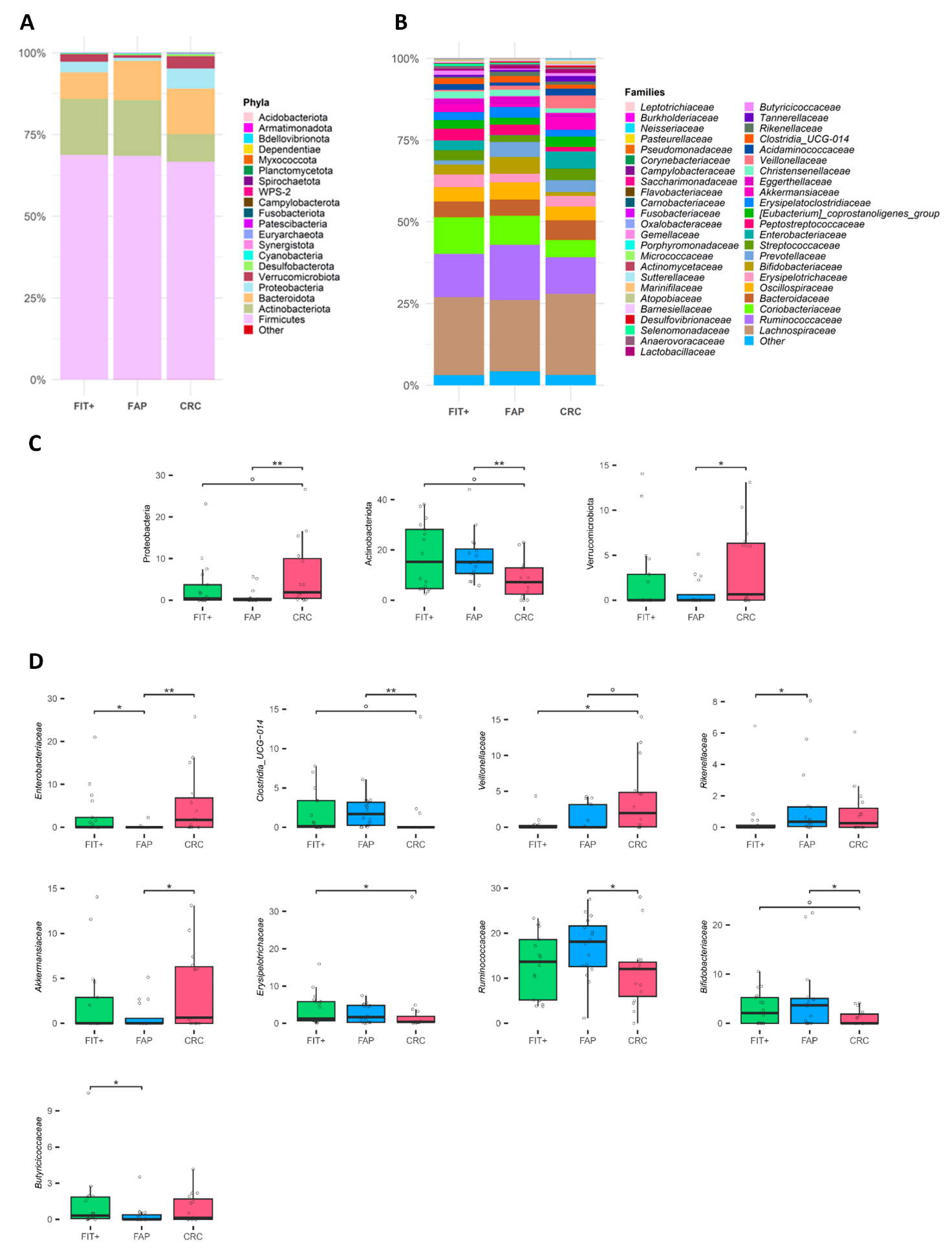


**Supplementary Figure 5. Phylum- and family-level compositional differences in the faecal microbiota between FAP, CRC and FIT+ subjects.** Relative abundance distribution of bacterial phyla (A) and families (B) differentially represented between groups. Relative abundance distribution of bacterial phyla (C) and families (D) differentially represented between groups. Wilcoxon test, ° *P* < .1; * *P* < .05; ** *P* < .01.


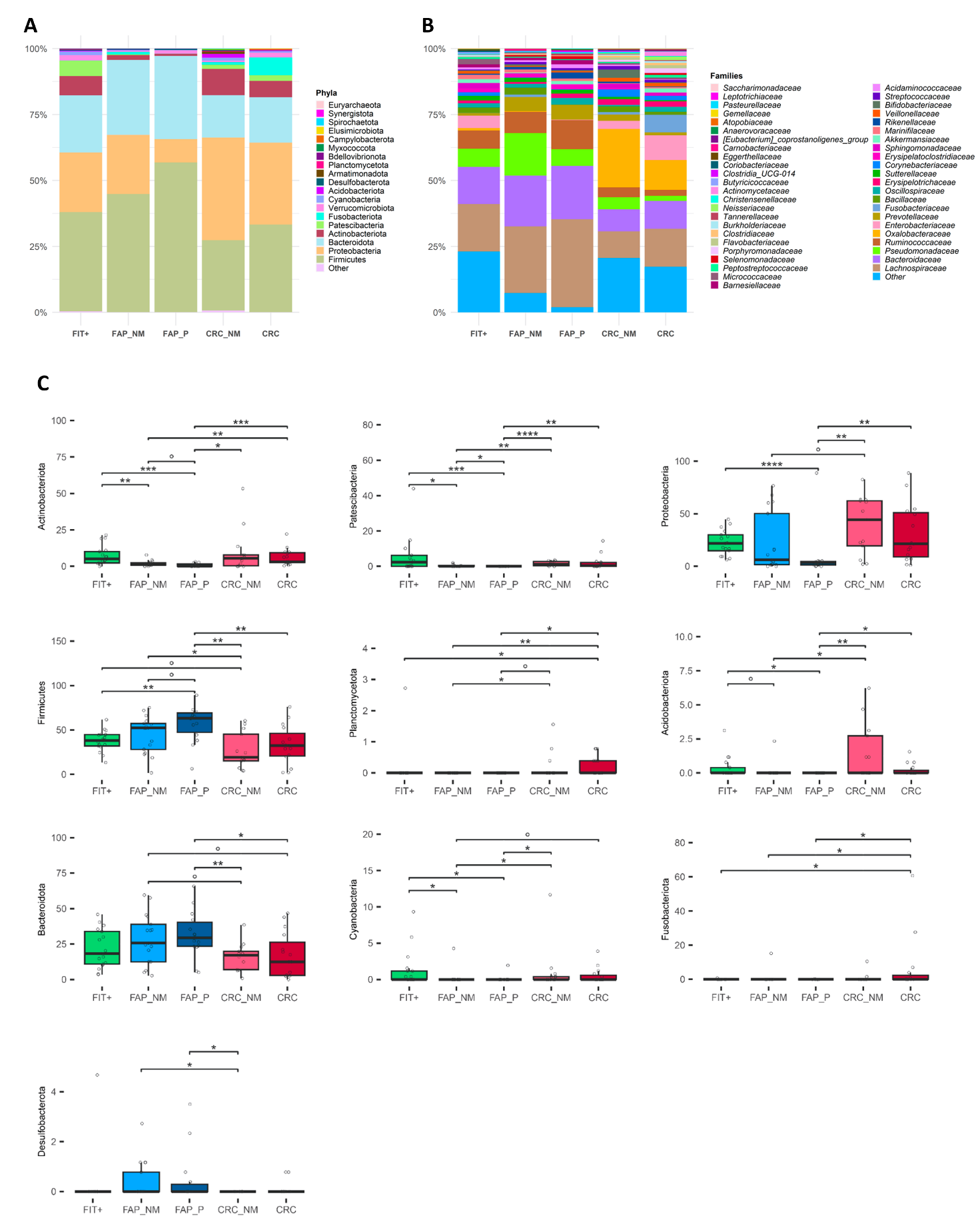


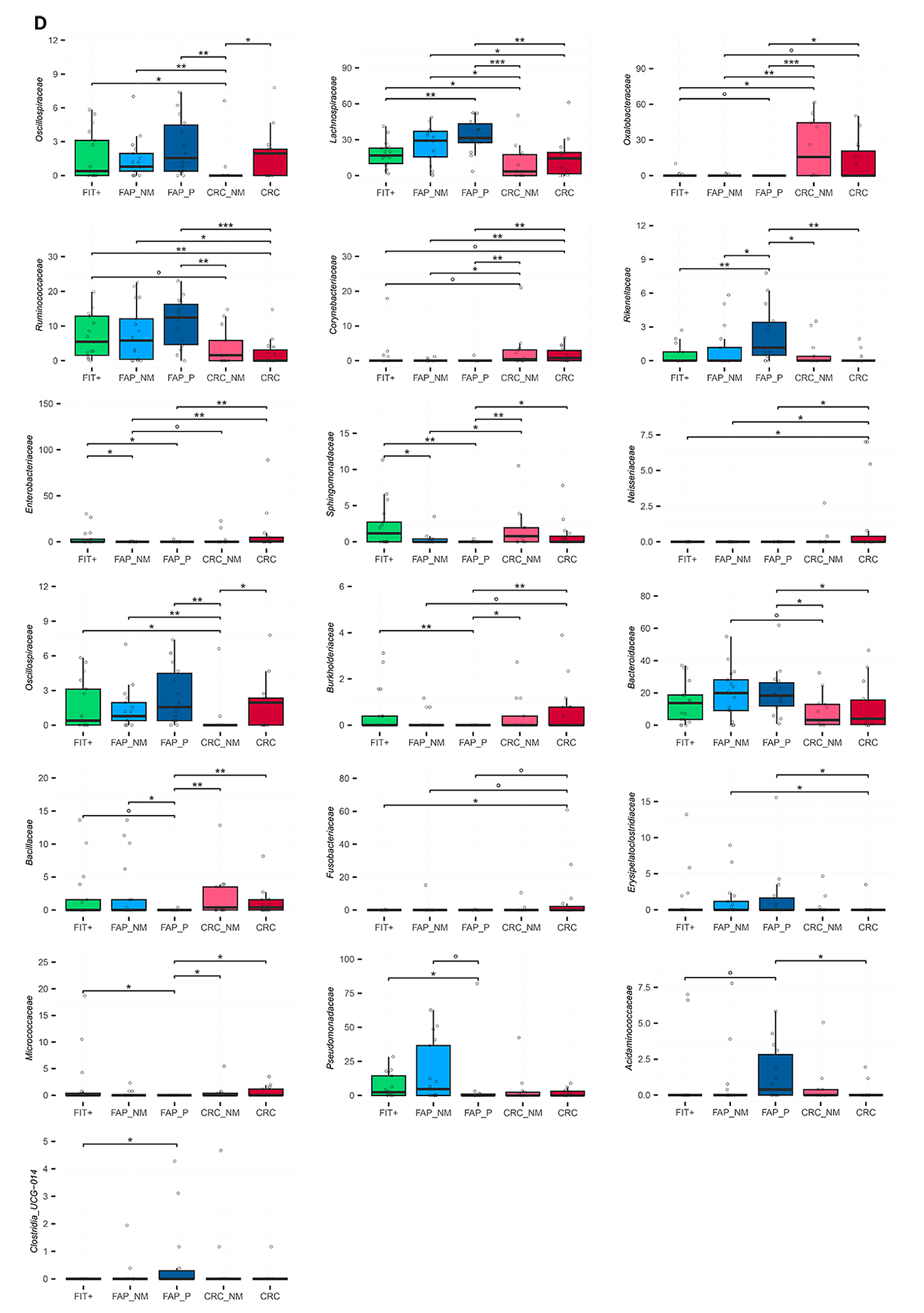


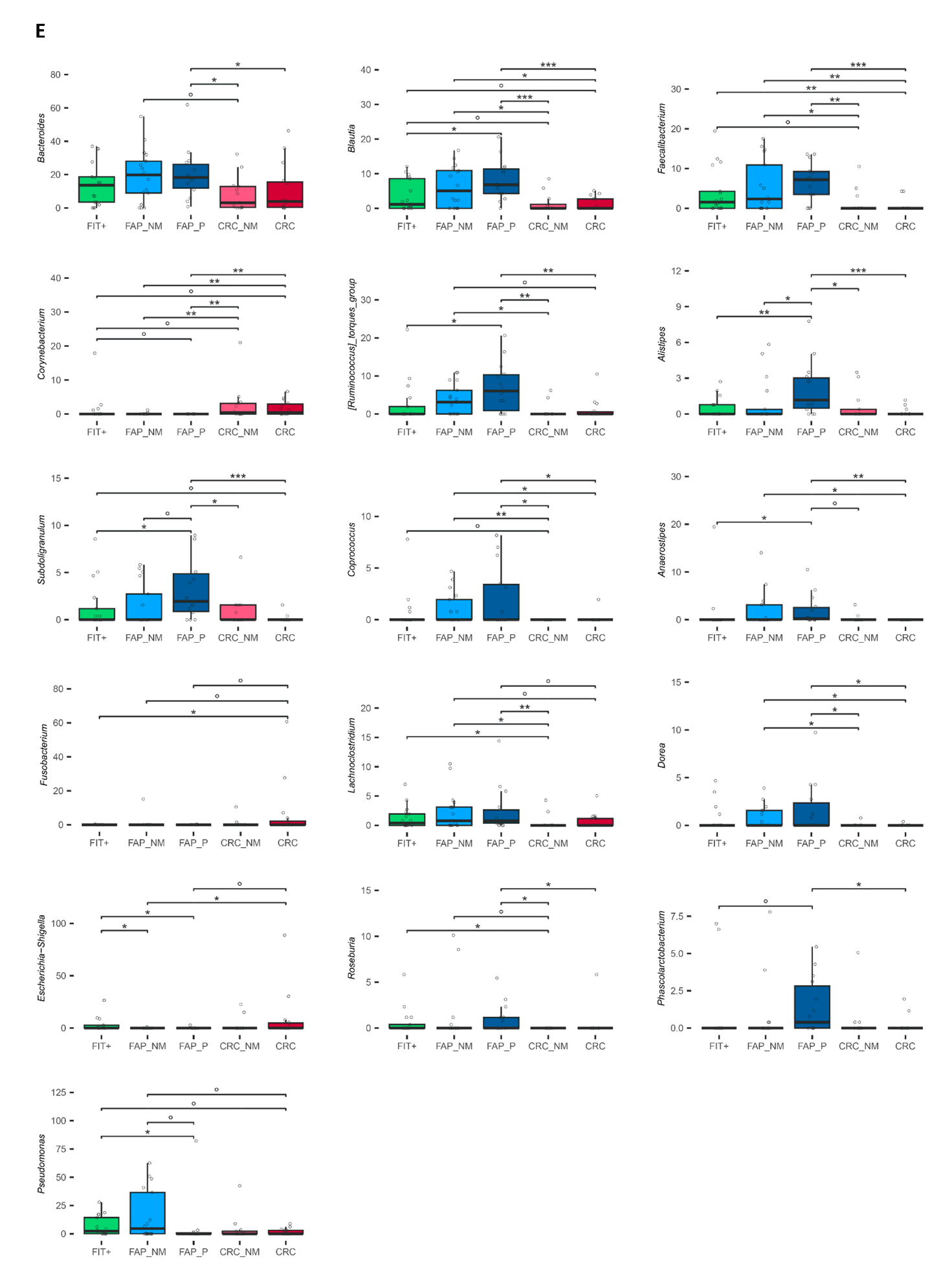


**Supplementary Figure 6. Phylum- and family and genus-level compositional differences in the mucosa-associated microbiota between FAP, CRC and FIT+ subjects.** Relative abundance distribution of bacterial phyla (A) and families (B). Relative abundance distribution of bacterial (C) phyla, (D) families and (E) genera differentially represented between groups (adenomatous polyps from FAP patients (FAP P), cancerous mucosa from CRC patients (CRC) and normal mucosa from FAP (FAP NM), CRC (CRC NM) and FIT+ subjects (FIT+). Wilcoxon test, ° *P* < .1; * *P* < .05; ** *P* < .01; *** *P* < .001.


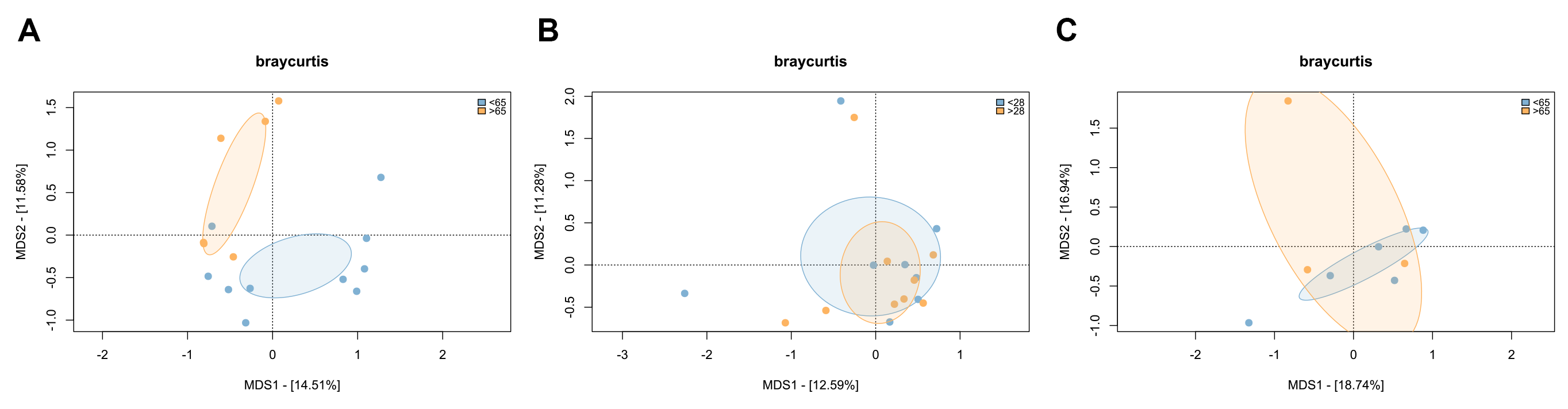


**Supplementary Figure 7. Age-related differences in the oral microbiota of FAP, CRC and FIT+ subjects.** Principal Coordinates Analysis based on Bray-Curtis dissimilarity between the oral microbiota profiles of FIT+ (A), FAP (B) and CRC (C) subjects stratified by age. Specifically, FIT+ and CRC subjects were stratified into adults and elderly using 65 years as the threshold. For the FAP group, which included no subjects over 65 years of age, the median age (28 years) was used to have two numerically comparable groups. Ellipses indicate a 95% confidence interval based on the standard error of the weighted average of sample coordinates. No significant segregation between age groups was found for any of the subject groups (Adonis *P* > .05)


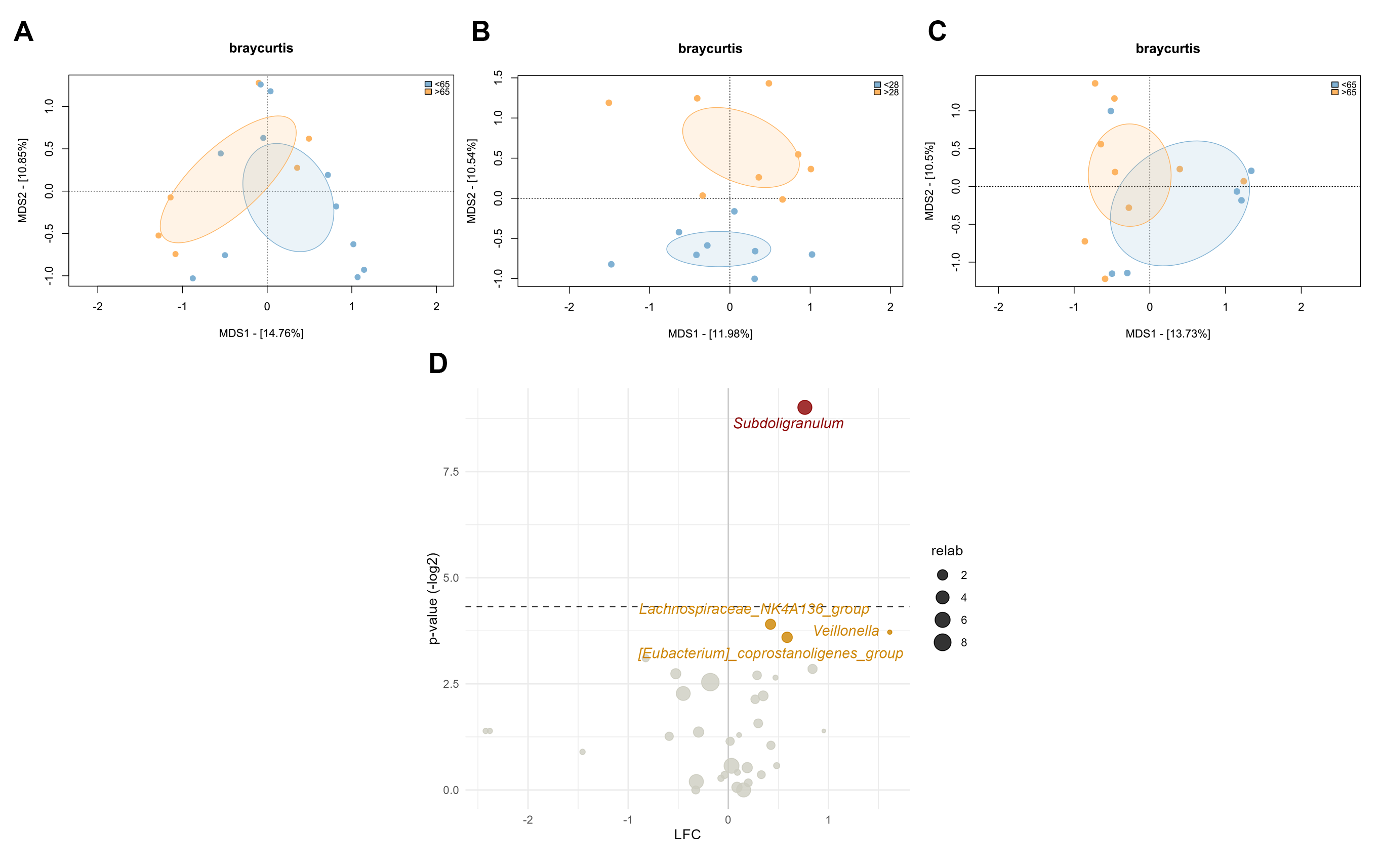


**Supplementary Figure 8. Age-related differences in the faecal microbiota of FAP, CRC and FIT+ subjects.** Principal Coordinates Analysis based on Bray-Curtis dissimilarity between the faecal microbiota profiles of FIT+ (A), FAP (B) and CRC (C) subjects stratified by age. Specifically, FIT+ and CRC subjects were stratified into adults and elderly using 65 years as the threshold. For the FAP group, which included no subjects over 65 years of age, the median age (28 years) was used to have two numerically comparable groups. Ellipses indicate a 95% confidence interval based on the standard error of the weighted average of sample coordinates. A significant segregation between the two age groups was found only for FAP patients (Adonis *P* = .048). (D) The volcano plot showing the main differences in the faecal microbiota composition of FAP patients stratified by age. The x-axis shows the natural logarithm of the fold change (LFC) in the relative abundance of genera between patients under and over 28 years. The y-axis shows the Wilcoxon *P* value, expressed as -

log2 of *P*. Red and orange dots indicate *P* < .05 and *P* < .1, respectively.


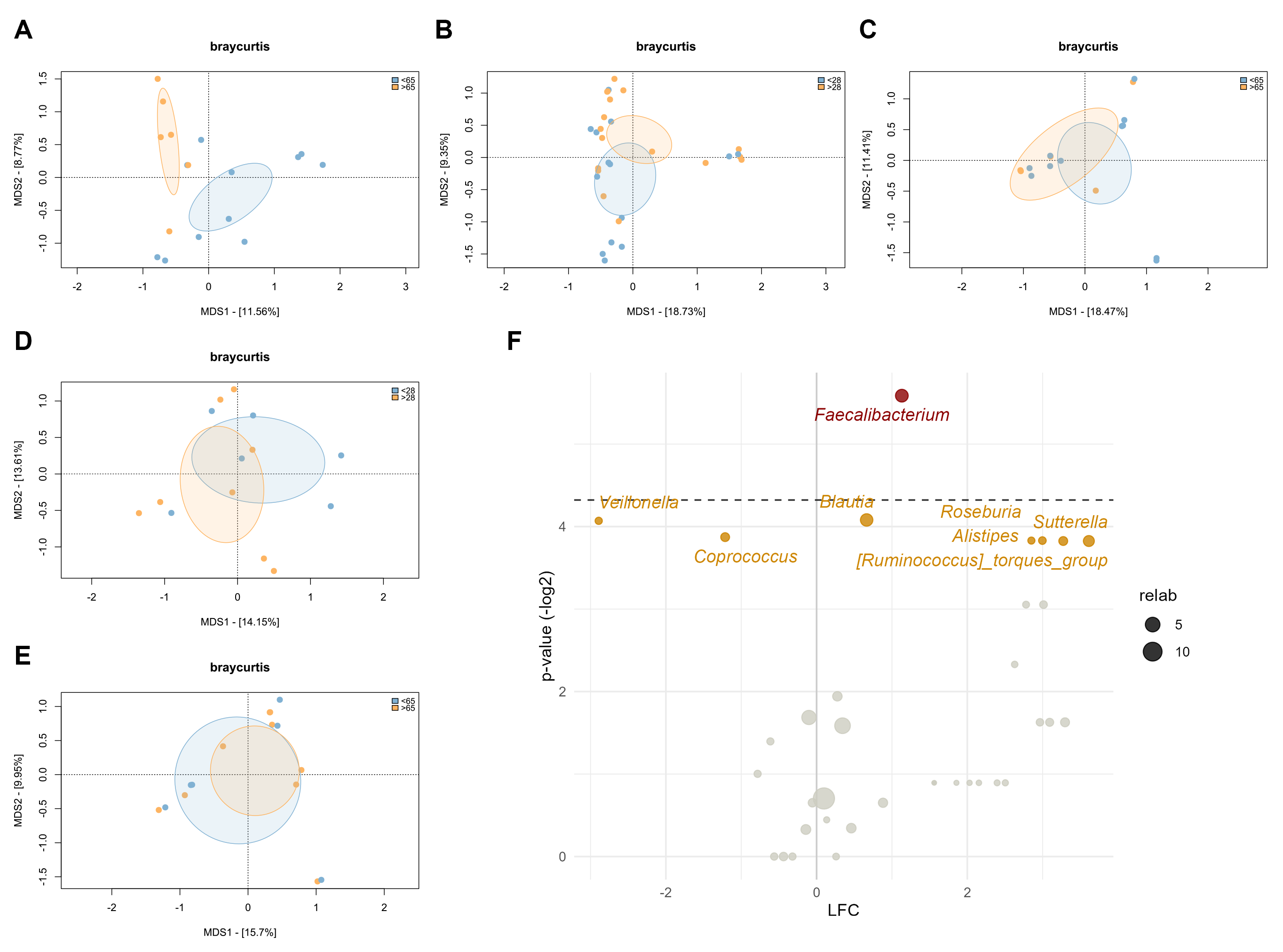


**Supplementary Figure 9. Age-related differences in the mucosa-associated microbiota of FAP, CRC and FIT+ subjects.** Principal Coordinates Analysis based on Bray-Curtis dissimilarity between the mucosa-associated microbiota profiles of FIT+ (A), FAP (B) and CRC (C) subjects stratified by age. Specifically, FIT+ and CRC subjects were stratified into adults and elderly using 65 years as the threshold. For the FAP group, which included no subjects over 65 years of age, the median age (28 years) was used to have two numerically comparable groups. Ellipses indicate a 95% confidence interval based on the standard error of the weighted average of sample coordinates. A significant segregation between the two age groups was found only for FIT+ subjects (Adonis *P* = .0065). The analysis was then repeated considering only adenomatous polyps for FAP patients (D) and the cancerous mucosa for CRC patients (E). No significant segregation was found (Adonis *P* > .54). (F) The volcano plot showing the main differences in the mucosa-associated microbiota composition of FIT+ subjects stratified by age. The x-axis shows the natural logarithm of the fold change (LFC) in the relative abundance of genera between patients under and over 65 years. The y-axis shows the Wilcoxon *P* value, expressed as -log2 of *P*. Red and orange dots indicate *P* < .05 and *P* < .1, respectively.

**
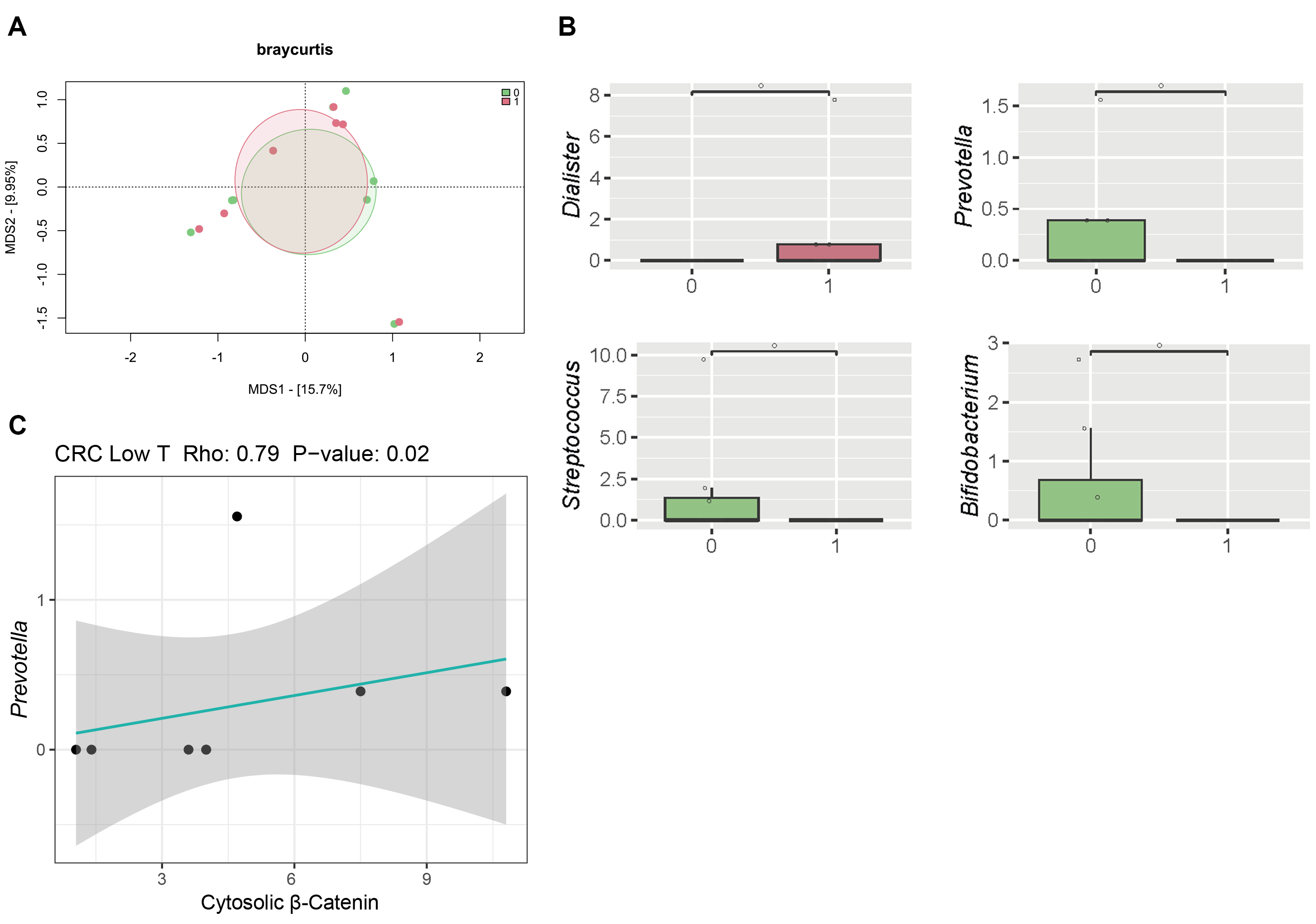
**

**Supplementary Figure 10. Correlation between mucosa-associated microbiota of CRC patients and disease severity, as based on the tumour-node-metastasis system.** (A) Principal Coordinates Analysis based on Bray-Curtis dissimilarity between the microbiota profiles of CRC patients stratified by pT stage into Low T “0” and High T “1” groups. No significant segregation was found (Adonis *P* = .95). (B) Boxplots showing the relative abundance distribution of genera that tended to be differentially represented between the two groups (Wilcoxon test, ° *P* < .1). (C) Spearman correlation between *Prevotella* and cytosolic β-catenin in the Low T group. Spearman’s rho and P are reported at the top of the plot.

**
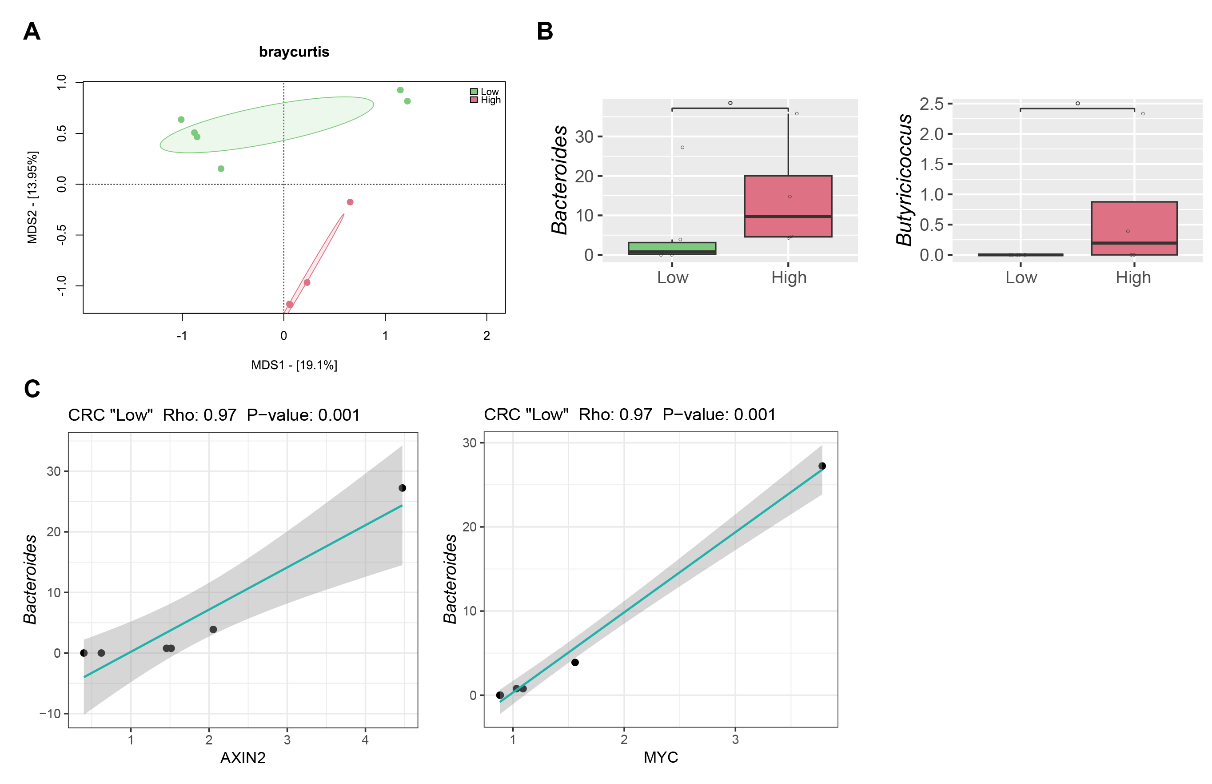
**

**Supplementary Figure 11. Correlation between mucosa-associated microbiota of CRC patients and immunoscore.** (A) Principal Coordinates Analysis based on Bray-Curtis dissimilarity between the microbiota profiles of CRC patients stratified by immunoscore (IS) into “Low” and “High” IS using the median value as a threshold. (B) Boxplots showing the relative abundance distribution of genera that tended to be differentially represented between the two groups (Wilcoxon test, ° *P* < .1). (C) Spearman correlation between *Bacteroides* and mTOR/WNT molecular biomarkers within the low IS group. Spearman’s rho and P are reported at the top of the plot.


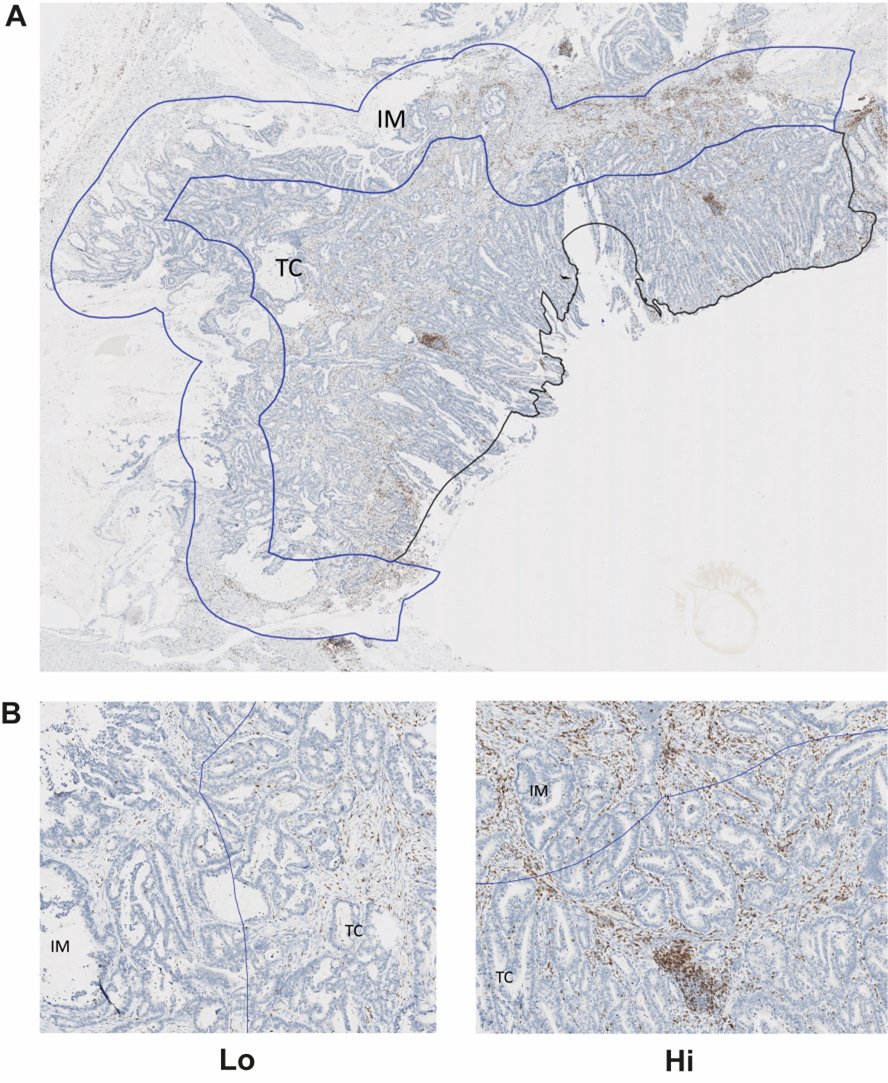


**Supplementary Figure 12.** A) Representative image of CD3+ staining in PT12 CRC tissue (original magnification, x10) showing the core of tumour (CT) and the invasive margin (IM) regions. B) Representative images (original magnification, x80) showing regions with low (Lo) and high (Hi) CD3+ cells density in PT12 CRC.
